# Hybridization and adaptive evolution of diverse *Saccharomyces* species for cellulosic biofuel production

**DOI:** 10.1186/s13068-017-0763-7

**Published:** 2017-03-27

**Authors:** David Peris, Ryan V. Moriarty, William G. Alexander, EmilyClare Baker, Kayla Sylvester, Maria Sardi, Quinn K. Langdon, Diego Libkind, Qi-Ming Wang, Feng-Yan Bai, Jean-Baptiste Leducq, Guillaume Charron, Christian R. Landry, José Paulo Sampaio, Paula Gonçalves, Katie E. Hyma, Justin C. Fay, Trey K. Sato, Chris Todd Hittinger

**Affiliations:** 1 Laboratory of Genetics, Wisconsin Energy Institute, J. F. Crow Institute for the Study of Evolution, Genome Center of Wisconsin, University of Wisconsin-Madison, Madison, WI USA; 2 DOE Great Lakes Bioenergy Research Center, University of Wisconsin-Madison, Madison, WI USA; 3 Microbiology Doctoral Training Program, University of Wisconsin-Madison, Madison, WI USA; 4 Laboratorio de Microbiología Aplicada, Biotecnología y Bioinformática, Instituto Andino Patagónico de Tecnologías Biológicas y Geoambientales, IPATEC (CONICET-UNComahue), Centro Regional Universitario Bariloche, Bariloche, Río Negro Argentina; 5 State Key Laboratory of Mycology, Institute of Microbiology, Chinese Academy of Sciences, Beijing, China; 6 Departement des Sciences Biologiques, Université de Montréal, Montreal, QC Canada; 7 Département de Biologie, PROTEO, Pavillon Charles-Eugène-Marchand, Institut de Biologie Intégrative et des Systèmes (IBIS), Université Laval, Quebec City, QC Canada; 8 UCIBIO-REQUIMTE, Departamento de Ciências da Vida, Faculdade de Ciências e Tecnologia, Universidade Nova de Lisboa, Caparica, Portugal; 9 Department of Genetics, Center for Genome Sciences and Systems Biology, Washington University in St. Louis, St. Louis, MO USA

**Keywords:** *Saccharomyces*, Biodiversity, Ammonia fiber expansion (AFEX), AFEX-pretreated corn stover hydrolysate (ACSH), Hybridization, Bioethanol, Xylose, Hydrolysate toxins

## Abstract

**Background:**

Lignocellulosic biomass is a common resource across the globe, and its fermentation offers a promising option for generating renewable liquid transportation fuels. The deconstruction of lignocellulosic biomass releases sugars that can be fermented by microbes, but these processes also produce fermentation inhibitors, such as aromatic acids and aldehydes. Several research projects have investigated lignocellulosic biomass fermentation by the baker’s yeast *Saccharomyces cerevisiae*. Most projects have taken synthetic biological approaches or have explored naturally occurring diversity in *S. cerevisiae* to enhance stress tolerance, xylose consumption, or ethanol production. Despite these efforts, improved strains with new properties are needed. In other industrial processes, such as wine and beer fermentation, interspecies hybrids have combined important traits from multiple species, suggesting that interspecies hybridization may also offer potential for biofuel research.

**Results:**

To investigate the efficacy of this approach for traits relevant to lignocellulosic biofuel production, we generated synthetic hybrids by crossing engineered xylose-fermenting strains of *S. cerevisiae* with wild strains from various *Saccharomyces* species. These interspecies hybrids retained important parental traits, such as xylose consumption and stress tolerance, while displaying intermediate kinetic parameters and, in some cases, heterosis (hybrid vigor). Next, we exposed them to adaptive evolution in ammonia fiber expansion-pretreated corn stover hydrolysate and recovered strains with improved fermentative traits. Genome sequencing showed that the genomes of these evolved synthetic hybrids underwent rearrangements, duplications, and deletions. To determine whether the genus *Saccharomyces* contains additional untapped potential, we screened a genetically diverse collection of more than 500 wild, non-engineered *Saccharomyces* isolates and uncovered a wide range of capabilities for traits relevant to cellulosic biofuel production. Notably, *Saccharomyces mikatae* strains have high innate tolerance to hydrolysate toxins, while some *Saccharomyces* species have a robust native capacity to consume xylose.

**Conclusions:**

This research demonstrates that hybridization is a viable method to combine industrially relevant traits from diverse yeast species and that members of the genus *Saccharomyces* beyond *S. cerevisiae* may offer advantageous genes and traits of interest to the lignocellulosic biofuel industry.

**Electronic supplementary material:**

The online version of this article (doi:10.1186/s13068-017-0763-7) contains supplementary material, which is available to authorized users.

## Background

Fossil fuel reserves are being depleted [1], and their use contributes to climate change [2]. To avoid the tremendous costs associated with continued fossil fuel consumption, renewable and clean energy sources must be developed to create a sustainable bioeconomy. Liquid transportation biofuels will be an important component of this new bioeconomy, and cellulosic bioethanol provides an attractive renewable energy source that is nearly CO_2_-neutral and compatible with much of the current distribution infrastructure [3]. Nonetheless, the bioconversion of lignocellulosic biomass to liquid fuels poses several challenges that have yet to be fully overcome [4].

*Saccharomyces* (*S.*) *cerevisiae* is the workhorse of the incipient lignocellulosic biofuel industry [5] due to its robustness, stress-tolerance compared to bacteria and other fermenting microbes [6], and the established infrastructure for production by the sugarcane and starch ethanol industries. Even so, the complex composition of lignocellulosic biomass [7] poses several specific challenges. First, hydrolysates made from lignocellulosic sources contain high levels of pentose sugars, particularly xylose, which native *S. cerevisiae* consumes poorly or not at all [8]. Second, these hydrolysates contain potent fermentation inhibitors that are mainly derived from the deconstruction of biomass during the chemical pretreatments used to improve the accessibility of cellulose and hemicellulose to hydrolysis [9]. For example, after enzymatic treatment and the application of the ammonia fiber expansion (AFEX) method used to deconstruct corn stover [10], phenolic amides, phenolic acids, furans, and other small inhibitory molecules are generated [11]; these molecules are collectively termed “hydrolysate toxins (HTs).” Proposed mechanisms for their toxicity include the inhibition of key enzymatic steps, such as glutamine PRPP amidotransferase (PurF), which is important for de novo purine biosynthesis but inhibited by feruloyl amide in *Escherichia coli* [12]; decreased energy availability due to costly efflux pumps [13]; and redox imbalances caused by the detoxification of acids and aldehydes [9].

Previous work has partially overcome the xylose conversion issue by introducing genes encoding efficient xylose metabolism enzymes into *S. cerevisiae*. For example, GS1.11-26 is a strain of *S. cerevisiae* derived from the corn ethanol industrial strain Ethanol Red. GS1.11-26 was engineered with the *Clostridium phytofermentans xylA* gene, which encodes xylose isomerase; cassettes to overexpress genes encoding enzymes of the pentose phosphate pathway; and several other genes of interest. Mutagenesis and adaptive evolution further improved xylose fermentation by GS1.11-26 [14]. Generally, this and similar strategies have focused on laboratory or corn ethanol strains of *S. cerevisiae* [15–19].

Our strategy has been to begin with one of the most stress-tolerant strains from a collection of wild *S. cerevisiae* strains (with pairwise nucleotide divergence values of up to nearly 0.8%), leading to the selection of *S. cerevisiae* NRRL YB-210, which was originally isolated from Costa Rican bananas [20–23]. NRRL YB-210 was then engineered with the genes *XYL1, XYL2,* and *XYL3* from *Scheffersomyces* (*Sch*.) *stipitis* [21], and it was evolved aerobically in rich media with xylose as the main carbon source, generating the strain GLBRCY73 (Y73) [24], whose heterothallic haploid derivative is GLBRCY101 (Y101). We have also previously described the engineering and evolution of GLBRCY128 (Y128). This haploid strain was also derived from NRRL YB-210, but it was engineered to overexpress *S. cerevisiae TAL1*, *C. phytofermentans xylA,* and *Sch. stipitis XYL3*, a strategy that bypassed the redox imbalance created by the *XYL1* and *XYL2* steps in Y73. This strain also underwent a series of adaptive evolution experiments in YPX, yielding a final strain that could ferment xylose anaerobically, including in AFEX corn stover hydrolysate (ACSH) [25, 26]. Even in these stress-tolerant, xylose-fermenting strains, the HTs in ACSH are still potent repressors of xylose fermentation [9].

New biological parts, including both genes and *cis*-regulatory elements, are critical for improving tolerance to HTs, biofuel production, and other traits of interest [3]. Unfortunately, de novo production of standardized, heterologous parts remains expensive, and predictions of which parts will achieve engineering goals remains limited by the scant knowledge of cellular networks [27]. The genus *Saccharomyces* includes six additional species beyond *S. cerevisiae* [28, 29]. These species are as genetically divergent at the protein sequence level as are humans and birds [30]. Diversity can be considerable even within a given *Saccharomyces* species. For example, *S. paradoxus* populations vary widely in freeze–thaw tolerance and temperature preferences [31], while different populations of *S. kudriavzevii* differ significantly in the gene content of their galactose utilization pathways [32]. European strains of *S. kudriavzevii* also have broad differences in aromatic compound production [33]. Thus, this genus offers an unparalleled opportunity to harness genetic variation for the improvement of biotechnological processes [29].

Hybridization among *Saccharomyces* species provides a facile method to combine traits, genes, and *cis*-regulatory elements [34, 35]. Several hybrids have been isolated from fermented beverages, such as wine, beer, and cider, which suggests that these hybrids are tolerant to stressful fermentative conditions and yield high-quality products with complex suites of traits that are difficult to obtain from a single species. [33, 36–45]. After several rounds of fermentation and transfers, new hybrids undergo genomic rearrangements, gene conversion, and gene copy number changes, presumably to maintain or amplify genes, from either parent, relevant to the industrial condition [40, 46, 47]. In some cases, these hybrids have been shown to outcompete both parents in the industrial condition, demonstrating heterosis (hybrid vigor) [48, 49].

In this work, we generated eight synthetic interspecies hybrids by crossing haploid engineered biofuel strains of *S. cerevisiae* with haploid derivatives of several *Saccharomyces* species, allowing us to explore whether hybridization is useful in biofuel research in a manner analogous to its use in the fermented beverage industry. Growth parameters were intermediate or showed evidence of heterosis. Six synthetic hybrids were evolved in ACSH microaerobically, exposing the cells to xylose and stress (e.g., starvation, ethanol toxicity). After selection, these evolved synthetic hybrids demonstrated improved fermentative properties, and genome sequencing of these strains revealed changes in the copy numbers of several chromosomal segments. To further explore the broad potential of the genus for biofuel production, we examined five hundred and seven wild, non-engineered *Saccharomyces* strains and found that several species and populations harbor genetic potential that could eventually be exploited for cellulosic biofuel production, such as HT tolerance and xylose consumption.

## Results

### Synthetic hybrids have intermediate traits or display heterosis

**Fig. 1:**
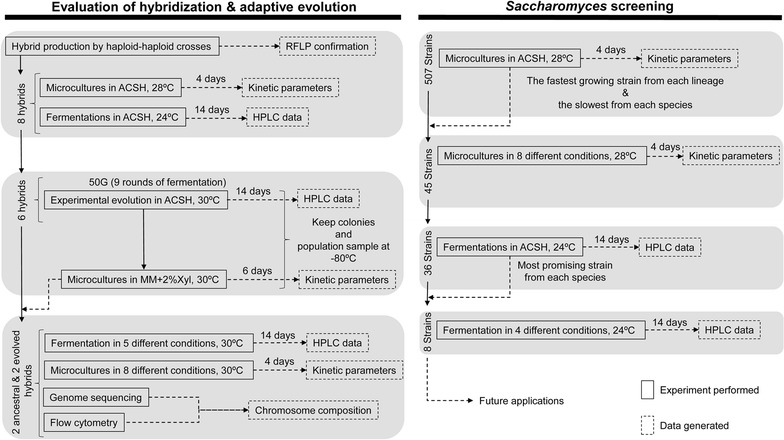
Schematic representation of the two main experiments. Three different temperatures were assayed. When working with wild isolates, we set the temperature of fermentation to room temperature (24 °C) or, for screening, the minimum allowed by the Tecan plate reader (28 °C). Adaptive evolution experiments and comparisons of the synthetic hybrids to Y73 were run at 30 °C, the temperature previously used to experimentally evolve Y73 [24]. *G* generations

To determine whether interspecies hybridization could introduce traits from other species relevant to biofuel production, we generated eight synthetic hybrids. Specifically, we crossed haploid strains of *S. cerevisiae* previously engineered and experimentally evolved for xylose fermentation (Y128 and Y101, a heterothallic haploid derivative of Y73) [24, 25] with previously generated haploid derivatives of the reference strains of *S. mikatae*, *S. kudriavzevii*, *S. uvarum* [28, 50], and our new heterothallic haploid derivative of *S. eubayanus* (Table 1; Fig. 1), four of the most divergent members of the genus.

**Table 1:** Haploid derivative, engineered strains, and ancestral and evolved hybrid strains information

Strain	Synonym	Parent	Species	Population	Genotype	Comment	Publication
Wild strains							
yHDPN14	FM1117		*Saccharomyces paradoxus*	A	*MAT* **a**/*MAT* **α**?; *ρ* ^*+*^ (C2_H69)		This study
yHAB336	SDPGP3		*Saccharomyces mikatae*		*MAT* **a**/*MAT* **α**?; *ρ* ^*+*^ (C2_H162)		This study
yHAB407	XXYS16L-5		*Saccharomyces kudriavzevii*		*MAT* **a**/*MAT* **α**?; *ρ* ^*+*^ (C2_H146)		This study
yHAB413	HZZt19L.1		*Saccharomyces arboricola*		*MAT* **a**/*MAT* **α**?; *ρ* ^*+*^ (C2_H158)		This study
yHCT77	FM1277		*Saccharomyces uvarum*	HOL-NA	*MAT* **a**/*MAT* **α**?; *ρ* ^*+*^ (C2_H77)		[60]
yHCT69	yHDPN9		*Saccharomyces eubayanus*	PB-HOL	*MAT* **a**/*MAT* **α**?; *ρ* ^*+*^ (C2_H97)		This study
Engineered strains							
GLBRCY73	yHDPN35		*Saccharomyces cerevisiae*		*MAT* **a**/*MAT* **α**; *ho*Δ::*XYL123*-*KanMX*; *ρ* ^*+*^ (C2_H13)	Evolved in YPX, aerobically	[24]
GLBRCY101	yHDPN47		*Saccharomyces cerevisiae*		*MAT* **a** *ho*Δ::*XYL123*-*KanMX*; *ρ* ^*+*^ (C2_H13)	Dissected from GLBRCY73	This study
GLBRCY128	yHDPN36		*Saccharomyces cerevisiae*		*ho*Δ::*ScTAL1*-*CpXylA*-*SchXYL3*-*KanMX*; *MAT* **a**; *ρ* ^*+*^ (C2_H13)	Evolved in YPX, anaerobically	[25]
yHSSS217		IFO1815^T^	*Saccharomyces mikatae*	Asia-A	*MAT* **α** *ho*Δ::*NatMX*; *ρ* ^*+*^ (C2_H74)		[28]
FM1097		IFO1802^T^	*Saccharomyces kudriavzevii*	Asia-A	*MAT* **α** *ho*Δ::*NatMX*; *ρ* ^*+*^ (C2_H83)		[28]
yHSSS101		CBS7001	*Saccharomyces uvarum*	HOL-EU	*MAT* **α** *ho*Δ::*NatMX*; *ρ* ^*+*^ (C2_H77)		[28]
yHEB69		FM1318	*Saccharomyces eubayanus*	PB-HOL	*MAT* **α** *ho*Δ::*TKMX*; *ρ* ^*+*^ (C2_H103)		This study
Synthetic hybrids							
yHDPN1	yHWA103		Y101 × *S. mikatae* yHSSS217		*MAT* **a**/*MAT* **α**; *ho*Δ::*XYL123*-*KanMX*/*ho*Δ::*NatMX*; *ρ* ^*+*^ (Recombinant)		This study
yHDPN5	yHWA93		Y101 × *S. kudriavzevii* FM1097		*MAT* **a**/*MAT* **α**; hoΔ::*XYL123*-*KanMX*/*ho*Δ::*NatMX*; *ρ* ^*+*^ (*S. cerevisiae*)		This study
yHDPN379			Y101 × *S. kudriavzevii* FM1097		*MAT* **a**/*MAT* **α**; hoΔ::*XYL123*-*KanMX*/*ho*Δ::*NatMX*; *ρ* ^*+*^ (*S. cerevisiae*)	yHDPN5 evolved in ACSH, microaerobically, 50 G	This study
yHDPN399			Y101 × *S. mikatae* yHSSS217		*MAT* **a**/*MAT* **α**; hoΔ::*XYL123*-*KanMX*/*ho*Δ::*NatMX*; *ρ* ^*+*^ (Recombinant)	yHDPN1 evolved in ACSH, microaerobically, 50 G	This study

Geographical, ecological, and genotypic information of selected *Saccharomyces* species for generating hybrids, or for conducting the three replicate fermentations in four media conditions at 24 °C, for wild isolates, or 30 °C for synthetic hybrids

C2_HXXX: Indicates the *COX2* haplotype based on [100]

*Sch*, *Scheffersomyces (Pichia) stipitis*; *Cp*, *Clostridium phytofermentans*; *Sc*, *Saccharomyces cerevisiae*; *G*, generations

*XYL123*, abbreviation for the *SchXYL1*-*SchXYL2*-*SchXYL3* cassette

**Fig. 2:**
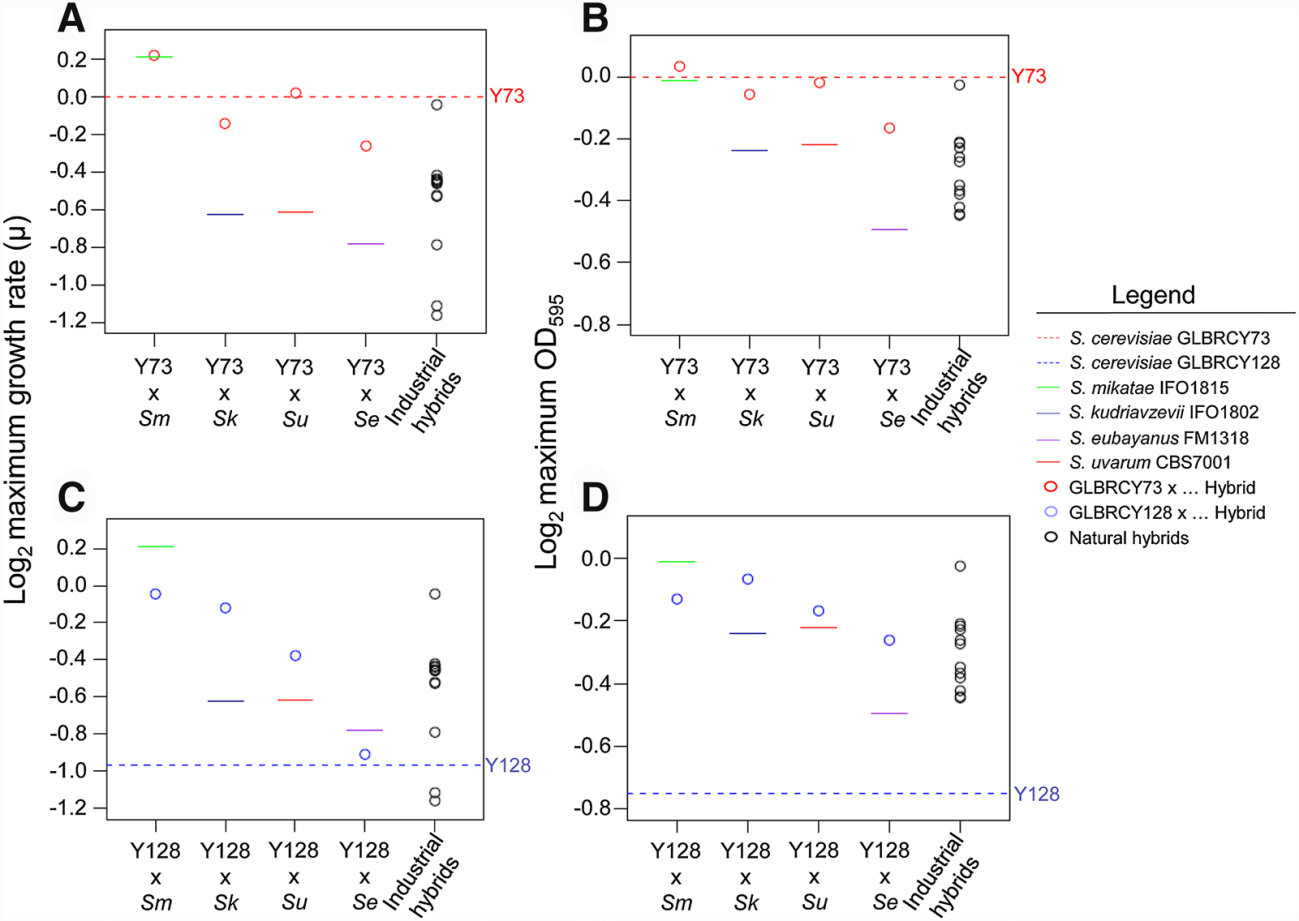
Synthetic hybrids have intermediate kinetic parameters or display heterosis compared to their parents during ACSH microtiter growth. The kinetic parameter averages (*n* = 2) of normalized values (see “Methods”) of maximum growth rate (*µ*, defined as (ln(OD_2_) − ln(OD_1_))/(*T*_2_ − *T*_1_)) and maximum OD_600_ from Y73 hybrids (**A**, **B**) and Y128 (**C**, **D**) are represented. By definition the normalized value of the reference Y73 strain is 0, and values above this threshold are better than the reference. Normalized values for Y128 are represented by a *dashed blue line*. Synthetic hybrids generated from crosses with the haploid derivatives Y73 and Y128 are represented by *red* and *blue dots*, respectively. Previously isolated synthetic hybrids from other industrial applications are represented by *black dots*. The values of the non-*S. cerevisiae* parents are represented by *colored lines* according to the legend. *Se*: *Saccharomyces eubayanus*, *Sk*: *Saccharomyces kudriavzevii*, *Sm*: *Saccharomyces mikatae*, *Su*: *Saccharomyces uvarum*

In microaerobic conditions (microtiter plates), Y128 grew more slowly than Y73 (*t* test, *p* value 2.379 × 10^−06^) (Fig. 2; Additional file 1), likely due to the disruption of key signaling pathways during the adaptation of Y128 to anaerobic growth in YPX media [25, 26] (Fig. 2). In general, hybrids isolated from fermented beverages grew more slowly than Y73 (*t* test, *p* value 1.551e−06) and the synthetic hybrids made with Y73 (*t* test, *p* value = 9.741e−05) (Fig. 2a). Thus, hybrid status itself does not enable better growth in ACSH; instead, the specific parents of the hybrids likely confer the relevant properties.

During growth in ACSH microtiter plates, the synthetic hybrids generally had intermediate kinetic traits relative to both parents (Fig. 2; Additional file 1). For example, Y73 × *S. mikatae*, Y128 × *S. mikatae*, and Y128 × *S. uvarum* grew faster than their *S. cerevisiae* parents (*t* test, *p* value <0.0010), while Y73 × *S. kudriavzevii* and Y73 × *S. eubayanus* grew faster than their non-*S. cerevisiae* parents (*t* test, *p* value <0.0300). One hybrid, Y128 × *S. uvarum*, showed significant heterosis by growing faster than both parents (*t* test, *p* value <0.0057). These data suggest that ACSH kinetic traits are heritable and, in many cases, at least partially dominant.

The synthetic hybrids retained the critical xylose consumption trait of their engineered *S. cerevisiae* parents, except for Y73 × *S. kudriavzevii*, whose extracellular xylose concentration remained unchanged after 14 days in culture at 24 °C (Additional file 2). Although the concentration of extracellular xylose decreased in most of the synthetic hybrids, the final concentration was higher than the *S. cerevisiae* parents (*t* test, *p* value = 0.0309). These results suggest that interspecies hybridization can generate phenotypic diversity, while often retaining important parental kinetic and fermentative traits.

### Adaptive evolution improved the fermentative traits of synthetic hybrids

To determine whether synthetic hybrids could be improved through adaptive evolution, six synthetic hybrids (those crossing Y73 or Y128 to either *S. mikatae*, *S. kudriavzevii,* or *S. uvarum*) were evolved for 50 generations in triplicate (Fig. 1). Each round consisted of fermentations in ACSH at 30 °C for 14 days under microaerobic conditions. Since glucose was exhausted by the second day, this stringent condition forced the cells toward starvation if they did not consume xylose, while simultaneously forcing them to retain tolerance to HTs and ethanol. Most of the synthetic hybrids did not survive these harsh conditions, and only two replicates of Y73 × *S. mikatae* and one replicate of Y73 × *S. kudriavzevii* reached the complete 50 generations of adaptive evolution. At the end of the adaptive evolution experiment, 10 colonies of each surviving hybrid were grown in Minimal Medium (MM) containing 2% xylose, and the fastest-growing variant was selected for further analysis.

**Fig. 3:**
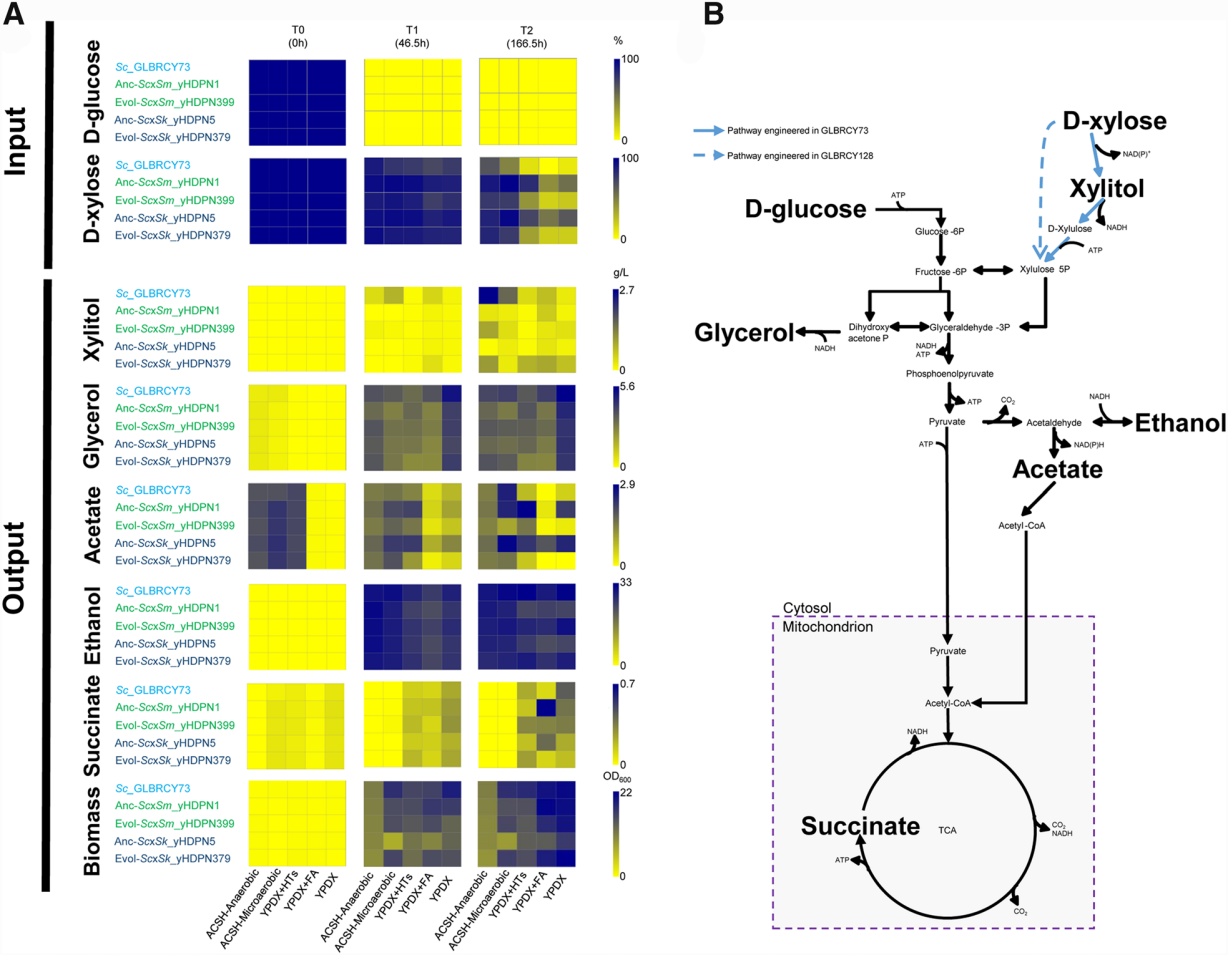
Evolved synthetic hybrids improved their fermentation traits during ACSH culture at 30 °C. Extracellular compound concentration averages (*n* = 3) for d-glucose, d-xylose, xylitol, glycerol, acetate, ethanol, and succinate, as well as OD_600_ for biomass, are represented by heatmaps. Heat colors from *yellow* (low value) to *blue* (high value) are scaled according to the bars in the right of each series of heatmaps. **B** Schematic representation of the metabolic pathway for glucose and xylose utilization. *Bold names* highlight the compounds shown in **A**. Continuous and discontinuous *blue arrows* indicate engineered steps in Y73 and Y128, respectively. *Sc*: *S. cerevisiae*, Anc: ancestral synthetic hybrid, Evol: synthetic hybrid evolved for 50 generations, *Sc*x*Sm*: *S. cerevisiae* × *S. mikatae*, *Sc*x*Sk*: *S. cerevisiae* × *S. kudriavzevii*, YPDX + FA: YPDX plus feruloyl amide, YPDX + LTs: YPDX plus cocktail of hydrolysate toxins (HTs). Heatmaps were generated from data in Additional file 4. Compound time course curves for xylose, glucose, ethanol, and biomass are also displayed in Additional files 6 and 7 for *S. cerevisiae* × *S. mikatae* and *S. cerevisiae* × *S. kudriavzevii* hybrids, respectively

**Fig. 4:**
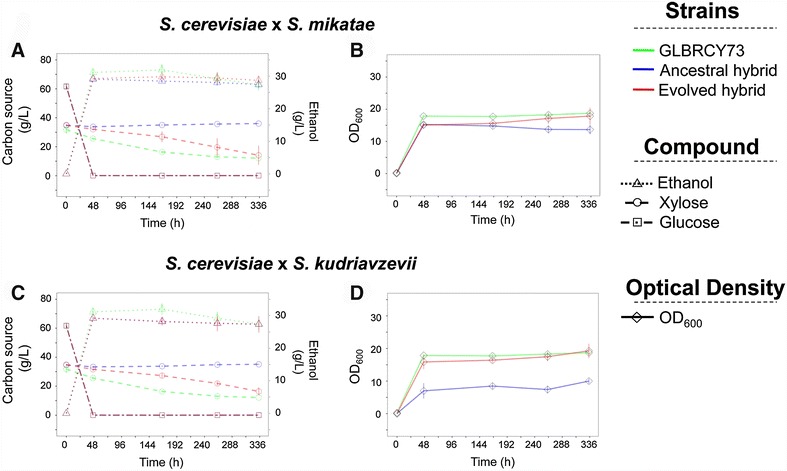
Time courses of ACSH microaerobic fermentations for GLBRCY73, synthetic hybrids, and evolved hybrids. **A**, **C** The extracellular concentration (g/L) of glucose, xylose, and ethanol through the fermentation in microaerobic conditions of ACSH at 30 °C for *S. cerevisiae* × *S. mikatae* and *S. cerevisiae* × *S. kudriavzevii,* respectively. **B**, **D** The variation of the optical density at 600 nm through the aforementioned fermentation

Next, we tested the growth rate of the ancestral synthetic hybrids and evolved hybrids in microtiter cultures we also examined metabolites of the glycolytic and pentose phosphate pathways (Fig. 3b; Additional file 4) during fermentations of the evolved hybrids, focusing on ACSH, YPDX (a rich medium containing glucose and xylose that matches their concentrations in ACSH), YPDX plus the HTs found in ACSH, and YPDX plus feruloyl amide (FA), a product of AFEX pretreatment previously shown to have inhibitory effects in *E. coli* [9, 12]. Despite lack of growth improvement in ACSH (Additional file 3), extracellular xylose concentrations in microaerobic ACSH fermentations decreased significantly faster in cultures of evolved hybrids than ancestral hybrids (*t* test, *p* value <0.0368) (Figs. 3, 4). The consumption of xylose translated into a slight but significant increase of ethanol during ACSH fermentation in the evolved hybrid Y73 × *S. mikatae*, as well as significant increases in other conditions for both evolved hybrids (Additional file 5). These results suggest that the evolved hybrids adapted to improve xylose fermentation at 30 °C in the presence of HTs, although respiration in the microaerobic conditions also likely occurred at later time points (Fig. 4). Other relevant traits included a significant reduction in extracellular concentrations of acetate and glycerol (*t* test, *p* value <0.0121) (Fig. 3).

To test the performance of our synthetic hybrids in more industrially relevant conditions, we also performed ACSH fermentations at 30 °C in anaerobic conditions for 7 days (Fig. 3; Additional files 6A, F, 7A, F). Xylose consumption and ethanol production in ACSH under anaerobic conditions remained qualitatively similar compared to microaerobic conditions. Interestingly, the extracellular xylose concentrations decreased faster in both evolved hybrids relative to ancestral hybrids (*t* test, *p* value <0.04886).

### Adaptive evolution drives genome rearrangements

Previous studies have shown that the genomes of interspecies hybrids are unstable [51–53]. To test whether our evolved hybrids experienced genome instability during their adaptation to ACSH, we sequenced their genomes, measured their relative DNA content by flow cytometry, and compared these data with that from their ancestors. As expected, ancestral hybrids were diploid, and the evolved Y73 × *S. mikatae* hybrid remained approximately diploid (Additional file 8). In contrast, flow cytometry and read coverage data indicated that the evolved hybrid Y73 × *S. kudriavzevii* was approximately triploid, suggesting an increase of the DNA content per cell (Additional file 8).

**Fig. 5:**
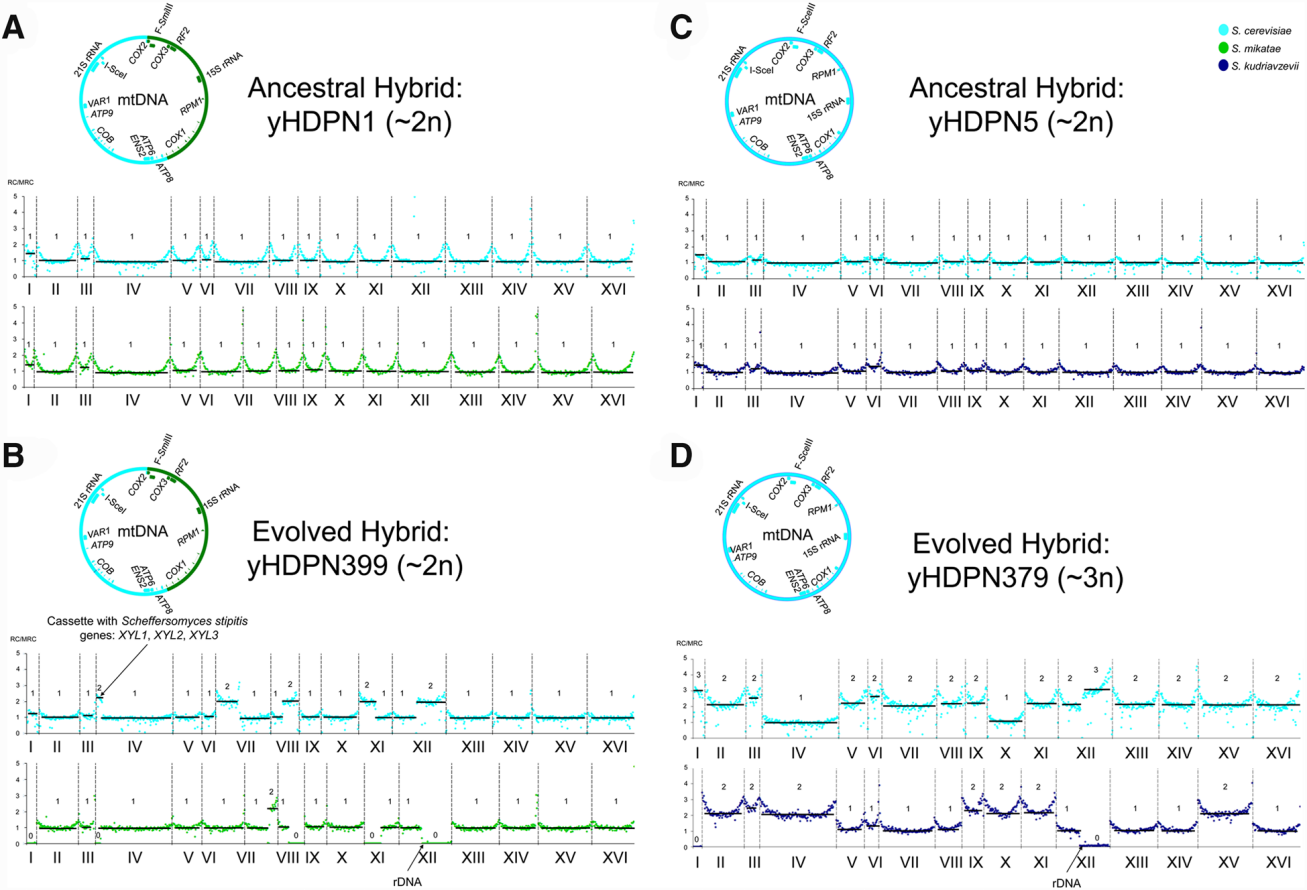
Adaptive evolution drives genome rearrangements during ACSH fermentation at 30 °C. Read coverage levels (RC), normalized using the median value of read coverage (MRC) for the complete genome, are shown for each subgenome of yHDPN1, yHDPN399, yHDPN5, and yHDPN379 in **A**–**D**). The levels were adjusted by establishing the lowest average coverage value for one copy. The genomic region where the *Sch. stipitis XYL1*, *XYL2*, and *XYL3* genes were inserted and the cluster of *r*ibosomal *DNA* are indicated. Approximate ploidy levels (Additional file 8) and chromosome content are included. Note that, in some libraries, the read mapping against a reference generates a “smiley pattern” where distal regions of chromosomes are better represented than proximal regions, a phenomenon for which chromatin structure may be responsible [99]. Mitochondrial genomes are displayed in *circles*, and the *color* corresponds to the species donor, denoted in the legend. Recombination in Y73 × *S. mikatae* was likely initiated in the *COX2* gene, a region previously described as a recombination hotspot due to the presence of the homing endonuclease gene F-*Sce*III [100]

Genome-wide ploidy levels were estimated using a reference-based coverage count of sequencing reads (Fig. 5). Chromosome copy numbers for both subgenomes in the ancestral hybrids followed the expected 1:1 ratio (Fig. 5a, c). However, both evolved hybrids contained gross chromosomal rearrangements, as suggested by increased read coverage for one of the parents and decreased read coverage for the other parent. The evolved hybrid of Y73 × *S. mikatae* contained interspecies translocations and copy number changes involving at least five chromosomes: IV, VII, VIII, XI, and XII. For example, the amplified left arm of Y73 chromosome IV in the evolved hybrid Y73 × *S. mikatae* contained the *Sch. stipitis* xylose utilization genes engineered into Y73 to confer xylose utilization, suggesting that these genes were amplified to increase their expression, while the homologous region of the *S. mikatae* genome was lost (Fig. 5b). The evolved hybrid of Y73 × *S. kudriavzevii* was mostly triploid, but several chromosomes were tetrasomic (2:2), including chromosomes II, III, V, VI, IX, XI, and XV. In both evolved hybrids, several non-*S. cerevisiae* genomic regions were replaced by their *S. cerevisiae* homologous counterparts (Fig. 5b, d). A translocation in chromosome XII was shared among both evolved hybrids, occurring ~50 Kb to the left of the cluster of *r*ibosomal *DNA* (*rDNA*). In both cases, the cluster of *rDNA* was inherited from *S. cerevisiae* subgenome. Thus, our stringent adaptive evolution experiment generated considerable genomic diversity, some of which might be involved in the fermentation improvements.

### Heritable hydrolysate tolerance of *S. mikatae* and other *Saccharomyces* strains

**Fig. 6:**
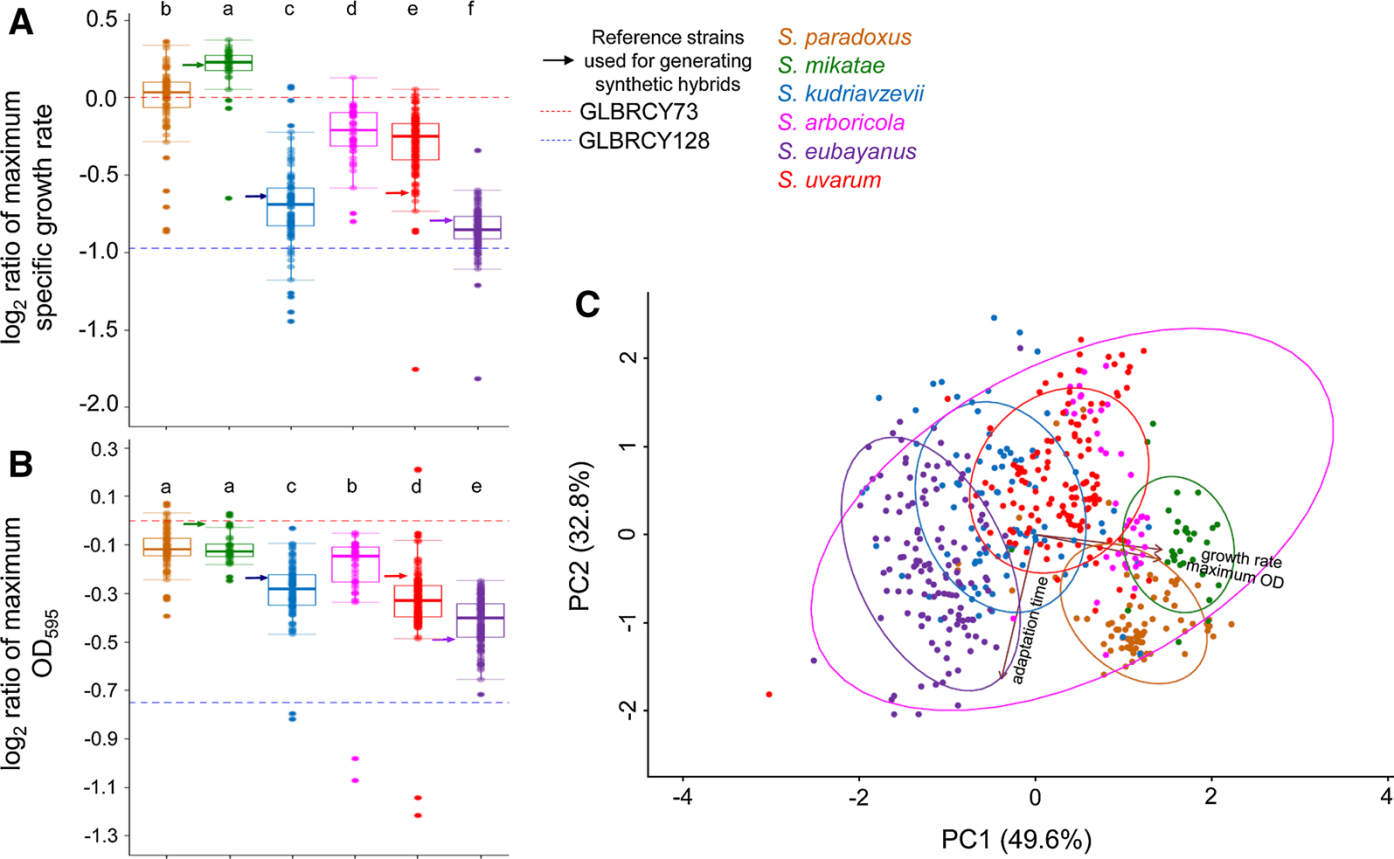
*S. mikatae* strains have innate tolerance to ACSH. **A**, **B** report the average (*n* = 2) of normalized maximum growth rate (*µ*, defined as (ln(OD_2_) − ln(OD_1_))/(*T*_2_ − *T*_1_)) and maximum OD_595_ for the 507 wild *Saccharomyces* strains. Median values for the species are represented by a *horizontal line* inside the box, and the *upper* and *lower whiskers* represent the highest and lowest values of the 1.5 * IQR (inter-quartile range), respectively. By definition, the values for the reference Y73 strain are 0, and values above this threshold are better than the reference. Normalized values for Y128 are represented by *dashed blue lines*. Letters are Dunn’s test homogeneous groups inferred from pairwise comparisons. Data points and* boxplots *are colored according to the species designation. *Arrows* highlight the values for the type strains, which were used to generate the synthetic hybrids. **C** Principal component analysis (PCA) summarizing three kinetic parameters for the wild *Saccharomyces* strains is shown. PC1 and PC2 accounted for 49.6 and 32.8% of the variation, respectively. *Color dots* represent the values for each *Saccharomyces* strain according to the legend. *Circles* indicate the clustering based on species designation. Variable weights are represented by *brown arrows*

Having shown that interspecies hybridization and adaptive evolution can generate diverse strains relevant to biofuel production, we interrogated whether genetically diverse strains of the various species of *Saccharomyces* might harbor additional potential (Fig. 1) for future research. We screened 507 wild strains of *Saccharomyces* from species other than *S. cerevisiae* and found a wide range of characteristics (Fig. 6A; Additional file 9). Notably, the kinetic parameters of ACSH microtiter cultures of the non-*S. cerevisiae* strains chosen as parents to construct the synthetic hybrids above were not necessarily the best strains for their respective species. These results suggest that additional hybridization experiments with diverse strains of *Saccharomyces* might unlock additional biofuel potential.

The *Saccharomyces* species differed significantly in their responses to ACSH (Kruskal–Wallis test *p* value <2.2 × 10^−16^) (Fig. 6A). A principal component analysis (PCA) demonstrated a strong correlation between maximum OD and growth rate among strains (Fig. 6C, Spearman’s correlation *ρ* = 0.67, *p* value = 2.2 × 10^−16^), while the length of the lag phase constituted a second principal component. *S. mikatae* and *S. paradoxus* produced more biomass and had faster maximum growth rates in ACSH than the other species (Fig. 6A, B). Although none of the species as a whole grew significantly faster than Y73 (Dunn’s test, corrected *p* value >0.05), whose genetic background was chosen specifically for ACSH tolerance [22], both species included several strains that grew faster (Fig. 6A). For example, *S. mikatae* IFO1815 grew faster than Y73 (*t* test, *p* value <9.7 × 10^−4^, Fig. 2a), a capability shared with many other *S. mikatae* strains. Indeed, *S. mikatae* grew significantly faster than the other *Saccharomyces* species (Dunn’s test, corrected *p* value <0.0455) (Fig. 6A), which suggests an innate tolerance to ACSH.

Several of our *Saccharomyces* strains have been classified into different populations or phylogenetic lineages (Additional file 9) [24, 25, 28, 32, 33, 43, 44, 54–63]. When we examined the kinetic parameters by splitting the strains by their lineage designation (Additional file 10), we found significant differences in growth rates between lineages for *S. paradoxus* and S*. eubayanus* (Kruskal–Wallis test, *p* value <0.05). For example, *S. paradoxus* strains from the America C (Québec) lineage [62] grew significantly faster than other lineages of this species (Dunn’s test corrected *p* value <0.0354). Similarly, strains from the Patagonia A and B lineages grew significantly faster than a set of closely related admixture strains (Dunn’s test corrected *p* value <0.0157), suggesting that this specific combination of alleles from these two lineages [33, 44] is detrimental in this particular condition. These associations further suggest that ACSH kinetic traits are heritable.

### Yeast diversity translates into fermentation trait diversity

**Fig. 7:**
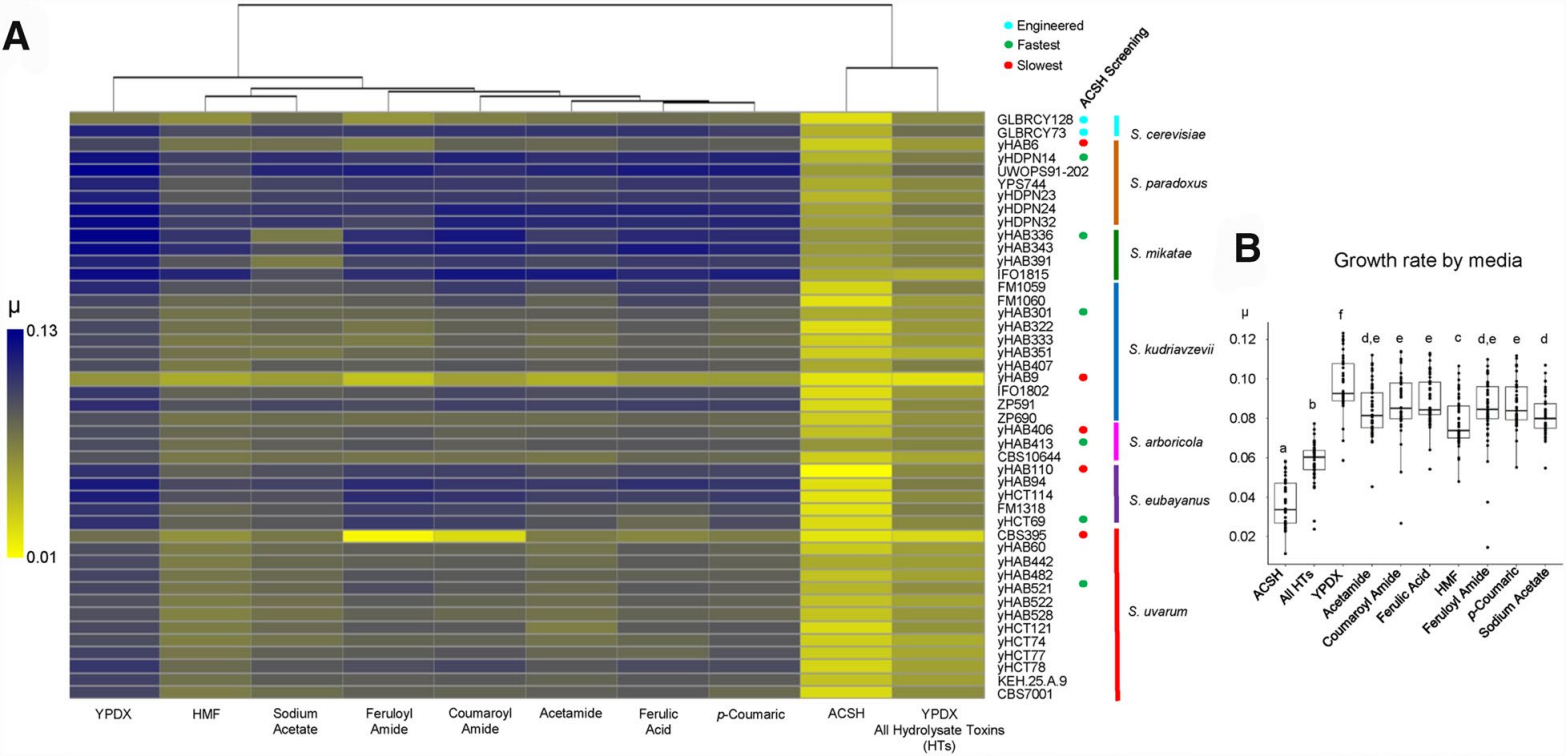
*Saccharomyces* strains differed in their responses to hydrolysate toxins. **A** The average values (*n* = 2) of maximum growth rate (*µ*, defined as (ln(OD_2_) − ln(OD_1_))/(T_2_ − T_1_)) in different media at 28 °C in 96-well plates are shown for the fastest-growing strain of each lineage, the slowest-growing strain of each species, the eight synthetic hybrids, and the two engineered *S. cerevisiae* reference strains. Heat colors from *yellow* (low growth rate) to *blue* (high growth rate) are scaled according to left bar. Engineered, best-growing, and worst-growing strains during ACSH screening are represented by *light blue*, *green*, and *red dots*, respectively. *Colored bars* represent the species designations. Media conditions are clustered by Euclidean distance. **B** Maximum growth rate boxplots by media condition. Median values for all media are represented by *horizontal lines* inside the boxes, and the *upper* and *lower whiskers* represent the highest and lowest values of the 1.5 * IQR (inter-quartile range), respectively. Letters are Dunn’s test homogeneous groups inferred after pairwise comparisons

To test the growth kinetics (microtiter cultures) in several inhibitory conditions, we selected the fastest- and slowest-growing strains of each species from the above screen (Additional file 9), as well as the fastest from each lineage (Fig. 1). Specifically, we tested ACSH, YPDX, and YPDX with various individual HTs (Fig. 7A; Additional file 11). In general, the *Saccharomyces* species had different responses to each condition (Kruskal–Wallis test *p* value <2.2 × 10^−16^). ACSH was the most inhibitory condition (Dunn’s test corrected *p* value <0.05), while YPDX plus a cocktail of HTs was the second most inhibitory (Fig. 7B). Although the latter was designed to mimic the effects of inhibitors found in ACSH, strains still grew an average of 47% more slowly in ACSH, further highlighting the chemical complexity of ACSH [11]. Strains growing in YPDX plus a single individual HT grew, on average, 9% more slowly than in YPDX without HTs (Fig. 7B). The best-growing strains of *S. paradoxus*, *S. mikatae*, *S. kudriavzevii*, and *S. arboricola* grew faster than other strains of their respective species for most individual HTs (Fig. 7A), suggesting that these strains bear novel genes or alleles that encode greater HT tolerance.

Individual HTs affected each species differently (Additional file 11). For example, compared to other *Saccharomyces* species, *S. mikatae* grew faster in most of the conditions testing individual HTs, except for in YPDX plus sodium acetate and YPDX plus the cocktail of HTs, which includes sodium acetate (Additional file 11). Thus, sodium acetate was the most inhibitory HT for our collection of *S. mikatae* strains (Dunn’s test, corrected *p* value = 0.0093). The kinetic parameters of strains were correlated among media, but the correlation values of the various media with ACSH and YPDX plus the cocktail of HTs were the lowest (Additional file 12).

**Fig. 8:**
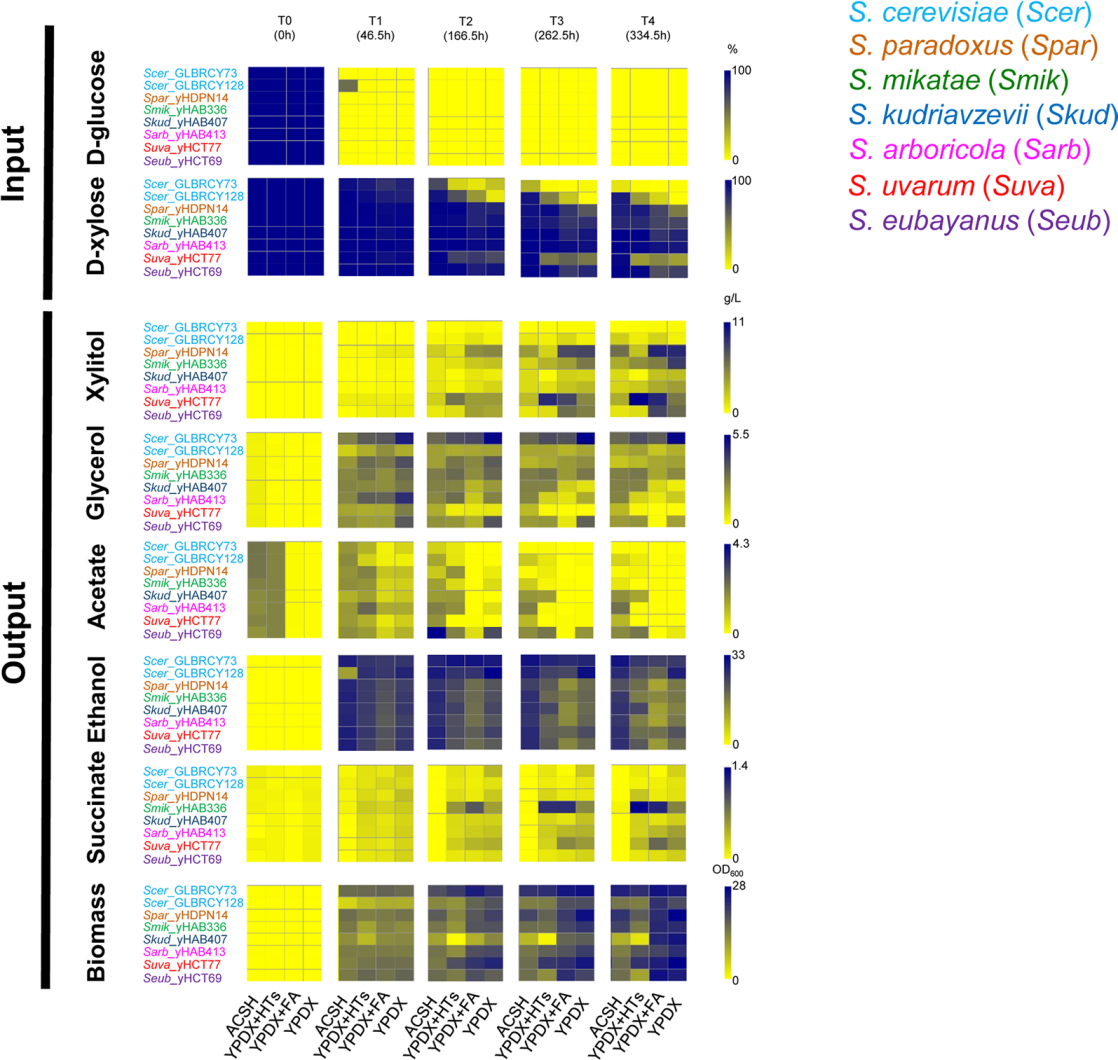
Xylose consumption and metabolite production by wild *Saccharomyces* strains. Extracellular compound concentration averages (*n* = 3) for d-glucose, d-xylose, xylitol, glycerol, acetate, ethanol, and succinate, as well as OD_600_ for biomass, are represented by heatmaps. Heat colors from *yellow* (low value) to *blue* (high value) are scaled according to the bars to the right of each compound. YPDX + FA: YPDX plus feruloyl amide, YPDX + LTs: YPDX plus cocktail of Hydrolysate Toxins (HTs). Heatmaps were generated from data in Additional file 4. Compound time course curves for xylose, glucose, ethanol, and biomass are also displayed in Additional file 13 for each individual strain

Next, we examined the fermentation traits of the selected strains (Additional file 9) in ACSH microaerobic fermentations at 24 °C (to ensure that even psychrophilic (cold-tolerant) strains grew well) for 14 days, measuring several key compounds, such as extracellular sugar and ethanol concentrations (Additional file 2). To determine how HTs specifically affected these fermentations, we then selected one wild strain of each non-*S. cerevisiae* species, together with Y73 and Y128 (Table 1), for additional fermentations (triplicate assays) in ACSH, YPDX plus a cocktail of HTs, YPDX plus feruloyl amide, and YPDX. In these triplicate fermentations, glucose was generally consumed within the first 48 h (Fig. 7; Additional files 4, 13). After glucose was consumed, many strains utilized xylose to some degree, mostly in non-ACSH media. Xylitol accumulation was highly correlated with decreased extracellular xylose (Spearman’s correlation *ρ* = −1, which is a rank-based test, *p* value = 0.0167) (Additional file 14A, B). Glycerol accumulated during the first 46 h (Additional file 14C), while acetate accumulated between 46 and 166 h (Fig. 8). In our microaerobic conditions, ethanol levels decreased for most of the strains after the second day (Additional file 14D), suggesting that biomass continued to be produced by the consumption of accumulated ethanol and glycerol (Additional file 14E).

As expected, the *S. cerevisiae* engineered strain Y73 was able to utilize xylose better than other *Saccharomyces* strains, except for in YPDX where Y73 and Y128 consumed similar levels (Wilcoxon rank sum test *p* value >0.05). Although xylose utilization was highly inhibited in ACSH compared to YPDX (Wilcoxon rank sum test, *p* value = 2.2 × 10^−16^), we were able to detect xylose utilization by other non-*S. cerevisiae* strains in other media (Fig. 8; Additional file 13). In particular, *S. uvarum* consumed significantly more xylose (Additional file 13G) than other non-*S. cerevisiae* strains in YPDX plus the cocktail of HTs and in YPDX (*t* test, corrected *p* value <0.0240), except when compared to *S. paradoxus* in YPDX medium (Additional file 13C), where they consumed xylose similarly.

## Discussion

### Interspecies hybridization and adaptive evolution combine and improve traits

Lignocellulosic biomass hydrolysates, such as ACSH, AFEX switchgrass hydrolysates, and wheat straw hydrolysates, are complex media [11, 64, 65] that pose particular challenges for efficient fermentations [9]. One method to begin to generate new strains with higher yields or tolerance is by selecting a chassis strain with useful properties and introducing biological parts from other organisms by genetic engineering [66]. However, this scheme requires specific knowledge of which biological parts are responsible for the traits of interest. Quantitative trait locus (QTL) mapping [67], comparative genomics [21, 68], chemical genomics [69], and several other methods have been successfully used to identify such parts, but each approach is labor intensive and costly.

Here we followed a rationale being applied in the fermented beverage industry, where several types of industrial hybrids are known, and researchers have recently constructed synthetic hybrids deliberately [70–72]. By crossing strains of *S. cerevisiae* engineered for xylose fermentation [21, 24] with different species of *Saccharomyces*, we efficiently combined their biological parts and, by adaptive evolution, established a selective regime where the hybrid strains adapted to ACSH while retaining hydrolysate tolerance and improving fermentative traits relative to the ancestral synthetic hybrids. Although none of these strains outperformed the Y73, repeating the process for additional generations may yield further improvements.

We detected several genomic rearrangements and copy number changes in these evolved hybrids, events frequently observed in *Saccharomyces* hybrids used in the fermented beverage industry [33, 40, 42, 43, 47, 73, 74]. At least some of these rearrangements and copy number changes probably confer adaptive properties, such as the increase in the number of copies of the *Sch. stipitis* xylose utilization (*XYL*) genes. Interestingly, the Y73 × *S. kudriavzevii* hybrid significantly improved its xylose consumption without amplifying these *XYL* genes, suggesting that its improved xylose utilization was driven by a distinct mechanism. Other rearrangements might be important for genome stability, such as the fixation of the *S. cerevisiae* cluster of *rDNA*, which is known to undergo concerted evolution in several domesticated yeasts [43, 75], as well as in interspecies hybrids in other types of organisms [76].

The genome content of hybrids from the fermented beverage industry ranges from approximately diploid to approximately tetraploid, and most strains are aneuploid [40, 47, 74]. Hybrids that are approximately triploid or tetraploid can be generated in two ways. The first model proposes haploid–haploid hybridization, followed by increases of ploidy, such as through endoreduplication [77]. A second model proposes so-called “rare mating” events where a diploid *MAT* **a** */MAT* **α** cell is converted to *MAT* **a** */MAT* **a** or *MAT* **α** */MAT* **α** by gene conversion and then mates with a haploid or another compatible diploid [46, 78]. Here one of our diploid synthetic hybrids increased its genome content to approximately triploid with several tetrasomic chromosomes. Although we cannot rule out a rare mating event in liquid culture, endoreplication followed by the losses of one of the four copies of most chromosomes seems to be the simplest explanation.

### *Saccharomyces* diversity as a source of biological innovation

*Saccharomyces* species offer a great opportunity to use interspecies hybridization to explore the vast genetic diversity present in the genus [30]. The likelihood that any natural strain would be optimal for all traits required for a particular industrial process is infinitesimal. Indeed, we found that none of the engineered strains, nor wild strains from the various *Saccharomyces* species were optimal for HT tolerance, xylose consumption, or ethanol production. This result highlights the importance of determining the genetic bases of industrially desirable traits so that they can be used to further engineer chassis strains for biofuel production.

Specifically, here we demonstrated that many strains of *S. paradoxus* and *S. mikatae* tolerate ACSH better than a strain of *S. cerevisiae* specifically chosen for its ACSH tolerance [24]. *S. paradoxus* was previously shown to tolerate furfural and hydroxymethylfurfural (HMF) [79, 80], results that our data replicate on a much broader strain collection. We also found that the under-characterized species *S. mikatae* might have even better tolerance traits than *S. paradoxus* for HMF and several other HTs. Interestingly, *S. paradoxus*, *S. mikatae*, and especially *S. uvarum* can also consume xylose to some extent in ACSH. In general, *Saccharomyces* have been found to not utilize xylose [81], but a handful of studies in *S. cerevisiae* [82, 83], together with this study on other *Saccharomyces*, have demonstrated that some strains do consume xylose, even though they do so slowly and mainly accumulate xylitol. Variation among strains in HT tolerance and xylose utilization could both be ecologically relevant. In nature, yeasts are likely to be differentially exposed to xylose and HTs, which can be derived from the degradation of lignocellulosic biomass by other fungi [84], deployed as defensive compounds by plants [85], or encountered in the guts of insects that feed on plants [86]. Thus, understanding the genetic basis of variation in these traits among yeast species and populations might simultaneously illuminate their ecologies and provide biotechnological benefits.

### Exploiting the vast genetic diversity of *Saccharomyces*

In this study, we chose *Saccharomyces* strains for hybridization prior to our comprehensive screen for traits of interest to biofuel production, in part because of the considerable labor required to generate stable haploid strains for crosses. To maximize the exploration of genetic traits from *Saccharomyces* species and allow crosses with asexual industrial strains, we recently developed the Hybrid Production (HyPr) system [72]. The utility of HyPr resides in its ability to generate allopolyploids by efficiently inducing rare mating between diploids and selecting for hybrids with reciprocal markers. With this methodology in hand, we can now easily generate allotetraploids and expose them to adaptive evolution to generate and recombine genomic diversity. Tetraploids may be of particular interest for these types of experiments because they have been shown to adapt faster than diploids and haploids [87]. Coupled with the identification of top performers for each *Saccharomyces* species, this new technology will allow us to explore a broader genomic landscape than we have done here with haploid–haploid synthetic hybrids, with the goal of ultimately determining the genetic basis of variation in traits relevant to biofuel production.

## Conclusions

The genus *Saccharomyces* offers an unparalleled opportunity to explore a vast range of genetic and phenotypic diversity with the aim of identifying key variants and biological parts for the improvement of xylose utilization, HT tolerance, and biofuel yield. Importantly, none of the wild isolates was the best performer for all desired fermentative traits. Since predicting desirable genetic variants and biological parts remains a major challenge, a protocol of interspecies hybridization and adaptive evolution offers a facile shortcut to recombine the genomes of new strains of interest with established biofuel chassis strains.

## Methods

### Yeast strains and maintenance

Our strain survey included five hundred and seven wild *Saccharomyces* strains, thirteen *Saccharomyces* hybrids isolated from fermented beverages, and eight synthetic hybrids. Strain information can be found in Additional files 1 and 9. As a standard reference, we used a strain of *S. cerevisiae* engineered and aerobically evolved for the consumption of xylose, Y73 [21, 24]. Yeast strains were stored in cryotubes with YPD (1% yeast extract, 2% peptone and 2% glucose) and 15% glycerol at −80 °C. Routine cultures were maintained in YPD plus 2% agar plates at 24 °C.

### Haploid-haploid crosses for hybrid production and confirmation

Haploid derivatives of *S. mikatae* IFO1815, *S. kudriavzevii* IFO1802, *S. uvarum* CBS7001 were previously generated [28]. Heterothallic haploid derivatives of Y73 (GLBRCY101) and *S. eubayanus* FM1318 (yHEB67) were generated in this study. For the generation of haploid derivative strains, the *HO* locus was replaced by a drug-resistance or *TK* [88] marker, and transformants were selected (Table 1). Sporulation was induced on sporulation plates (1% Potassium Acetate, 0.1% yeast extract, 0.05% glucose, 2% agar) or YPD at 24 °C, and tetrads were microdissected and micromanipulated using the SporePlay Dissector (Singer Instruments, UK). The strain of *S. cerevisiae* engineered and anaerobically evolved for the consumption of xylose, Y128 [25], is a haploid strain, so sporulation was not required. Mating type was determined by crossing the *hoΔ*::*DominantMarker* haploid strains with diagnostic auxotrophic haploid strains that were *MAT* **a** or *MAT* **α**. Successful crosses were able to grow in minimal medium (MM: 0.67% Yeast Nitrogen Base, 0.1% monosodium glutamate, 2% glucose) plus the conditions required to select for the dominant marker.

Heterothallic haploid Y101 or Y128 strains were each crossed with four haploid, *MAT*-compatible, non-*S. cerevisiae* strains. After pre-culturing strains at 24 °C overnight in YPD liquid, *MAT*-compatible cells were cultured in a 1.5-mL Eppendorf tube and patched in YPD agar plate to allow mating. After 12–36 h of incubation, a small amount of the patch was transferred to a YPD plate with the two corresponding drugs or other selective cocktails. DNA from successful growing colonies was extracted by the NaOH method [63, 89]. The hybrid nature of the nuclear genome was tested by a PCR-based random fragment length polymorphism (RFLP) method [42], and parental mitochondrial contribution was confirmed by PCR and Sanger-sequencing the mitochondrial *COX2* gene using primers previously described [90]. Eight synthetic hybrids were isolated and stored in cryotubes with YPD plus 15% glycerol at −80 °C.

### Microtiter plate growth curves

Our complete collection of strains was pre-cultured in deep 96-well plates with 500 µl of synthetic complete (SC: 0.67% Yeast Nitrogen Base without amino acids and without ammonium sulfate, 0.079% Drop-out Mix Complete, 0.5% ammonium sulfate, 2% glucose) until saturation at 24 °C. After pre-culture, 10 µl of saturated culture was inoculated into a 96-well plate (Nunc, Roskilde, Denmark) containing 240 µl of ACSH. Outer wells contained 250 µl of sterile water and were not inoculated. Each plate contained the reference strain Y73. Growth curve analysis and kinetic parameter estimation are detailed below.

Based on the results of this initial screen, the fastest-growing strain of each species or lineage and the slowest-growing strain of each species were selected, resulting in a total of forty-five *Saccharomyces* strains that also included the type strains of each species, the reference biofuel chassis strains, and eight synthetic hybrids (Fig. 7). The strain of *S. mikatae* chosen as its slowest-growing representative was discovered to not be a strain of *Saccharomyces* during the study, so it was removed from further analysis; all other strains were confirmed to be correctly identified. These representative strains are stored in a unique deep 96-well plate with YPD plus 15% glycerol. We calculated growth kinetic parameters in YPDX (1% yeast extract, 2% peptone, 6% glucose and 3% xylose), YPDX plus a cocktail of HTs (80 mM acetamide, 5.5 mM coumaroyl amide, 0.71 mM ferulic acid, 5.5 mM feruloyl amide, 1.1 mM hydroxy-methyl-furfural, 2.1 mM *p*-coumaric acid, and 32 mM sodium acetate), YPDX plus each individual HT (at concentrations indicated in the recipe for YPDX plus the cocktail of HTs). All media were adjusted to pH 5.2 with HCl. The concentrations of HTs are based their estimated concentrations in ACSH, previously published as Synthetic Hydrolysate v2 (SynH2) [11]. Feruloyl and coumaroyl amide were synthesized as described previously [11]. Each plate contained five copies of the reference strain Y73.

To monitor the growth of strains in the different conditions, inoculated 96-well plates were placed in Tecan F500 (Tecan Trading, Männedorf, Switzerland) maintaining an interior temperature set to a minimum of 28 °C. Selected synthetic ancestral and evolved hybrids (see below) and GLRBCY73 were tested at 30 °C (Fig. 1). Absorbance at 595 nm was monitored every 15 min for 5 days. Background absorbance was subtracted from the average of three negative controls (media without cells). Kinetic parameters for each condition were calculated in GCAT [91]. Average and standard deviations from two independent biological replicates were calculated in R [92]. Kinetic parameter values from the first ACSH screen were normalized against values from the reference strain, Y73.

### Fermentations and extracellular compound measurement

ACSH was prepared following the previously described method [93] with autoclaving prior to enzymatic hydrolysis, and its approximate composition has been determined [11]. Exploratory ACSH fermentations of selected strains (Fig. 1; Additional file 9), *S. paradoxus* strain CBS432, and eight synthetic hybrids were performed in 50 mL of ACSH at 24 °C in microaerobic conditions (250-mL flasks with airlocks) to minimize the sensitivity to higher temperatures of psychrophilic *Saccharomyces* species, such as *S. eubayanus*, *S. kudriavzevii*, and *S. uvarum* [94]. The starting OD at *λ* = 600 nm was 0.2, and shaking was performed at 110 rpm. A 600-µl sample from each individual flask was collected from fourteen time points: 0, 22.5, 46.5, 70.5, 94.5, 118.5, 142.5, 166.5, 190.5, 214.5, 238.5, 262.5, 286.5, and 334.5 h. Absorbance at 600 nm was measured using a DiluPhotometer spectrophotometer (Implen, Munich, Germany). Samples were centrifuged at 15,000 rcf for 1 min to pellet cells. Supernatant was analyzed in high-performance liquid chromatography (HPLC, see below) to measure extracellular compounds.

Six wild non-*S. cerevisiae* strains, one from each species, were selected based on their kinetics and potential xylose consumption (Table 1). These promising strains, along with the two engineered *S. cerevisiae* strains, were selected for more detailed fermentations in ACSH, YPDX, YPDX plus a cocktail of HTs (see above), and YPDX plus 1.2 mM feruloyl amide at 24 °C for 14 days. Three biological replicates were performed in independent 150-mL flask (see above) experiments a week or more apart. Similar fermentations were performed for *S. cerevisiae* Y73, two synthetic hybrids, and two evolved hybrids (see below), except that they were performed at 30 °C. Samples from five time points (0, 46.5, 166.5, 262.5, and 334.5 h) were processed as above for absorbance and extracellular compounds measurements.

To assess performance of key strains in a more industrially relevant condition, anaerobic fermentations were performed in ACSH at 30 °C for Y73, two synthetic hybrids, and two evolved hybrids. Media and cultures were degassed in an anaerobic chamber where O_2_ levels remained below 30 ppm. In this case, we sampled each day during the 7 days of fermentation.

Extracellular acetate, d-glucose, d-xylose, ethanol, glycerol, succinate, and xylitol in ACSH, YPDX and derivatives were determined by HPLC (Agilent 1260 infinity) using a quaternary pump, chilled (4 °C) autosampler, vacuum degasser, and refractive index detector (Agilent Technologies, Inc., Palo Alto, CA). The HPLC column consisted of an Aminex HPX-87H column (Bio-Rad) operating at 50 °C, a mobile phase of 0.02N H_2_SO_4_, and a flow rate of 0.5 ml/min.

### Adaptive evolution

Six of the synthetic hybrids (those generated between crosses of GLRBCY101 (heterothallic haploid derivative of Y73) or Y128 with haploid derivatives of *S. mikatae*, *S. kudriavzevii*, and *S. uvarum*) were evolved in triplicate for 50 generations at 30 °C in ACSH in microaerobic conditions (shake flasks with airlocks). 14 days of fermentation were performed to allow cells to consume xylose, and an aliquot of each replicate was transferred to a fresh ACSH until it reached 50 generations. The absorbance of the starting cultures and pitching density was 0.2 OD at *λ* = 600 nm, and OD was monitored at the end of each fermentation to calculate the number of generations per round of fermentation. When necessary, we performed sample dilutions to stay in the linear range of the spectrophotometer. From each round and replicate, three samples of 1 ml of the population culture were stored at −80 °C. From fermentation round 5 to the end of adaptive evolution experiment, a sample from each individual replicate was cultured on YPD plates to isolate 10 colonies. Colonies were stored at −80 °C. When the experiments reached 50 generations, the 10 colonies were pre-cultured overnight in SC plus 0.2% glucose, and 10 µl was transferred into a 96-well plate with 240 µl of MM plus 2% xylose (Table 1) to select the fastest-growing hybrid variant for further analyses.

### DNA content and genome sequencing

DNA contents of ancestral and evolved hybrids were determined by flow cytometry as previously described [72]. The diploid Y73 (2n) and the tetraploid W34/70 (4n) were used as references.

Paired-end Illumina HiSeq2000 platform 2 × 100 reads were generated (Additional file 15). Reads were demultiplexed, and adapters were removed using Trimmomatic [95], with parameters: 2:30:10 TRAILING:3 MINLEN:25. Species genome contributions to the ancestral and evolved synthetic hybrids were estimated as described previously [33] using genome assemblies of *S. cerevisiae*, *S. mikatae*, and *S. kudriavzevii* downloaded from www.saccharomycessensustricto.org [28]. Illumina reads were deposited in the SRA public database of NCBI under BioProject accession number SRP090190. Mitochondrial inheritance was assessed by assembling the synthetic hybrid genomes using SPAdes [96] as implemented in the wrapper iWGS v1 [97]. Scaffolds with a GC content lower than 30% were selected and mapped against the *S. cerevisiae* ancestor of the GLRBCY strains, Y22-3 [23], *S. mikatae* (accession number KX657747), and *S. kudriavzevii* (accession number KX657746) mitochondrial genomes, using Geneious R6 [98].

### Statistical analysis

A non-parametric Kruskal–Wallis test was applied to infer significant differences among data by using a Dunn’s test, which also conducts an ad hoc test for pairwise comparisons. *P* values were corrected for multiple comparisons to control the false discovery rate (FDR), by applying the Benjamini-Hochberg correction. Values were considered significant when the corrected/adjusted *p* value was below the FDR threshold of 5%. Correlation tests were conducted using the non-parametric Spearman’s rank correlation test. The reported *t* test was approached as an unpaired *t* test because independent samples were assayed for significance. Statistics, Principal Component Analysis (PCA), boxplots, and other graphs were conducted in R (R Development Core Team 2010).

## Additional files

**Additional file 1.** Geographical, genetic, and kinetic parameter information for hybrids.

**Additional file 2.** Initial fermentation screen to select the most-promising *Saccharomyces* strains. An exploratory, single replicate fermentation was conducted, and the extracellular metabolite concentrations of selected wild *Saccharomyces* and synthetic hybrids, ethanol yields based on sugar consumed, and percentages of glucose and xylose at the end of the 14 days ACSH fermentation at 24 °C are given. Strain names in green were selected for the next round of triplicate fermentations in four different media conditions at 24 °C. 1: selected engineered strain, 2: selected based on kinetics, 3: selected based on fermentative traits. Green, red, and gray row background colors indicate the best growing strains, the slowest growing strains, and the synthetic hybrids, respectively. Glu: glucose; Xyl: xylose; EtOH: ethanol; Gly: glycerol; Xli: xylitol; Ace: acetate; Cons: consumed.

**Additional file 3.** Maximum growth rate heatmap of two ancestral and two evolved synthetic hybrids and the GLBRCY73 strain. A) The average values (n = 2) of maximum growth rate (µ, defined as (ln(OD_2_)-ln(OD_1_))/(T_2_-T_1_)) in different media conditions at 30 °C are shown. Heat colors from yellow (low growth rate) to blue (high growth rate) are scaled according to left bar. Media conditions are clustered by Euclidean distance. *Sc*: *S. cerevisiae*, *Sm*: *S. mikatae*, *Sk*: *S. kudriavzevii*.

**Additional file 4.** Mean and standard deviation (SD) of extracellular compounds during fermentations at different time points.

**Additional file 5.** Ethanol yield (%) for ancestral synthetic hybrids and evolved synthetic hybrids during the xylose fermentation phase of the culture. Bar plots represent the difference between the percentage of ethanol yield at day seven and day 2, the point at which all glucose had been consumed. Panel A and B represent the values for *S. cerevisiae* × *S. mikatae* and *S. cerevisiae* × *S. kudriavzevii* synthetic hybrids, respectively. *S. cer*, *S. cerevisiae*; *S. mik*, *S. mikatae*; *S. kud, S. kudriavzevii.* Colors are the values for each condition according to the legend. ACSH-Anaer, ACSH anaerobic; ACSH-Micro, ACSH microaerobic; YPDX + HTs, YPDX + Hydrolysate toxins cocktail; YPDX + FA, YPDX + Feruloyl Amide. The *p*-values from t-tests are represented by * when < 0.05, ** < 0.01, *** < 0.001.

**Additional file 6.** Compound time course curves for GLBRCY73, synthetic ancestral hybrids, and evolved hybrids of *S. cerevisiae* × *S. mikatae*. Panels A-E represent the extracellular concentration (g/L) of glucose, xylose, and ethanol through the fermentation of various media and conditions for *S. cerevisiae* × *S. mikatae*. Panels F-J represent the variation of the optical density at 600 nm through the aforementioned fermentations.

**Additional file 7.** Compound time course curves for GLBRCY73, synthetic ancestral hybrids, and evolved hybrids of *S. cerevisiae* × *S. kudriavzevii*. Panels A-E represent the extracellular concentration (g/L) of glucose, xylose, and ethanol through the fermentation of various media in various conditions for *S. cerevisiae* × *S. kudriavzevii*. Panels F-J represent the optical density at 600 nm variation through the aforementioned fermentations. *S. cer, S. cerevisiae; S. kud, S. kudriavzevii.*

**Additional file 8.** Flow cytometry fluorescence intensity distribution for reference strains and synthetic hybrids. Y73 is a diploid strain [21, 24], and W34/70 is a hybrid between *S. cerevisiae* and *S. eubayanus* that is approximately tetraploid [101]. By comparing the cell count distribution of fluorescence intensity of SYBR green of each strain, we infer strains yHDPN1, yHDPN5, and yHDPN399 to be approximately diploid (2n). The yHDPN379 distribution is between Y73 and W34/70 distribution, suggesting that it is approximately triploid (3n). *Scer*: *S. cerevisiae*, *Smik*: *S. mikatae*, *Skud*: *S. kudriavzevii*, *Seub*: *S. eubayanus*. A.U.: Arbitrary Units.

**Additional file 9.** Genetic and kinetic parameter information for *Saccharomyces* strains.

**Additional file 10.** Normalized maximum growth rate, maximum OD_595_, and lag time boxplots by populations. The average values (n = 2) of normalized maximum growth rate (µ, defined as (ln(OD_2_)-ln(OD_1_))/(T_2_-T_1_)), maximum OD_595_, and lag time (adaptation time, h) for strains designated in lineages (Additional File 5) are shown. Median values for each lineage are represented by a black horizontal line inside the box, and the upper and lower whiskers represent the highest and lowest values of the 1.5 * IQR (inter-quartile range), respectively. Letters are Dunn’s test homogeneous groups inferred after pairwise comparisons. Colored lines highlight the values for the type strains, which were used to generate the synthetic hybrids.

**Additional file 11.** Maximum specific growth rates for each species and synthetic hybrids in specific media conditions. The average values (n = 2) of maximum growth rate (µ, defined as (ln(OD_2_)-ln(OD_1_))/(T_2_-T_1_)), from data represented in Fig. 6 but categorized by each condition and by species, are shown as boxplots. Letters are Dunn’s test homogeneous groups inferred from pairwise comparisons. Colored boxplots and data points are according to the legend. Median values for each population are represented by a horizontal line inside the box, and the upper and lower whiskers represent the highest and lowest values of the 1.5 * IQR (inter-quartile range), respectively. HTs: hydrolysate toxins.

**Additional file 12.** Pairwise correlation heatmaps for each media tested at 24 °C. A), B), and C) represent the pairwise Spearman correlation heatmaps among media tested in Fig. 6. Heat colors represent the degree of correlation from blue (low correlation among strain response, 0) to red (high correlation among strain response, 1). HTs: hydrolysate toxins. µ: (ln(OD_2_)-ln(OD_1_))/(T_2_-T_1_).

**Additional file 13.** Compound time course curves for engineered *S. cerevisiae* and wild *Saccharomyces* strains. Panels A-H represent the extracellular concentration (g/L) of glucose, xylose, and ethanol through microaerobic ACSH fermentation by different engineered and wild *Saccharomyces* strains. Panels I-P represent the variation of the optical density at 600 nm through the aforementioned fermentations.

**Additional file 14.** Compound dynamics through fermentation in different media conditions. Averages and standard deviations of D-xylose, xylitol, glycerol, ethanol concentration, as well as biomass production during the fermentations reported in Fig. 7 are shown in A), B), C), D), and E) graph plots, respectively. Colored lines represent the media condition according to the legend. FA: ferulic acid, HTs: hydrolysate toxins.

**Additional file 15.** Summary of whole genome sequencing statistics.

## Abbreviations

ACSH: AFEX-pretreated corn stover hydrolysate
AFEX: ammonia fiber expansion
A.U.: arbitrary units
FDR: false discovery rate
HMF: hydroxy-methyl-furfural
HPLC: high-performance liquid chromatography
MM: minimal medium
OD: optical density
PCA: principal component analysis
PRPP: 5-phosphoribosyl-1-pyrophosphate
RFLP: random fragment length polymorphism
SC: synthetic complete
YNB: yeast nitrogen base

## References

[1] British Petroleum (2016). BP statistical review of world energy.

[2] Intergovernmental Panel on Climate Change (2015). Climate Change 2014: mitigation of climate change.

[3] Liao JC, Mi L, Pontrelli S, Luo S (2016). Fuelling the future: microbial engineering for the production of sustainable biofuels. Nat Rev Microbiol.

[4] Sims REH, Mabee W, Saddler JN, Taylor M (2010). An overview of second generation biofuel technologies. Bioresour Technol.

[5] Klinke HB, Thomsen AB, Ahring BK (2004). Inhibition of ethanol-producing yeast and bacteria by degradation products produced during pre-treatment of biomass. Appl Microbiol Biotechnol.

[6] Olsson L, Hahn-Hägerdal B (1996). Fermentation of lignocellulosic hydrolysates for ethanol production. Enzyme Microb Technol.

[7] Jin M, Sarks C, Gunawan C, Bice B, Simonett S, Narasimhan RA, Willis L, Dale B, Balan V, Sato T (2013). Phenotypic selection of a wild *Saccharomyces cerevisiae* strain for simultaneous saccharification and co-fermentation of AFEX™ pretreated corn stover. Biotechnol Biofuels.

[8] Jeffries TW (2006). Engineering yeasts for xylose metabolism. Curr Opin Biotechnol.

[9] Piotrowski JS, Zhang Y, Bates DM, Keating DH, Sato TK, Ong IM, Landick R (2014). Death by a thousand cuts: the challenges and diverse landscape of lignocellulosic hydrolysate inhibitors. Front Microbiol.

[10] Lau MW, Dale BE (2009). Cellulosic ethanol production from AFEX-treated corn stover using *Saccharomyces cerevisiae* 424A(LNH-ST). Proc Natl Acad Sci USA.

[11] Keating DH, Zhang Y, Ong IM, McIlwain S, Morales EH (2014). Aromatic inhibitors derived from ammonia-pretreated lignocellulose hinder bacterial ethanologenesis by activating regulatory circuits controlling inhibitor efflux and detoxification. Front Microbiol.

[12] Pisithkul T, Jacobson TB, O’Brien TJ, Stevenson DM, Amador-Noguez D (2015). Phenolic Amides are potent inhibitors of de novo nucleotide biosynthesis. Appl Environ Microbiol.

[13] Pampulha ME, Loureiro-Dias MC (2000). Energetics of the effect of acetic acid on growth of *Saccharomyces cerevisiae*. FEMS Microbiol Lett.

[14] Demeke MM, Foulquié-Moreno MR, Dumortier F, Thevelein JM (2015). Rapid evolution of recombinant *Saccharomyces cerevisiae* for xylose fermentation through formation of extra-chromosomal circular DNA. PLoS Genet.

[15] Karhumaa K, Wiedemann B, Hahn-Hägerdal B, Boles E, Gorwa-Grauslund MF (2006). Co-utilization of -arabinose and d-xylose by laboratory and industrial *Saccharomyces cerevisiae* strains. Microb Cell Fact.

[16] Ha SJ, Galazka JM, Kim SR, Choi JH, Yang X, Seo JH, Glass NL, Cate JHD, Jin YS (2010). Engineered *Saccharomyces cerevisiae* capable of simultaneous cellobiose and xylose fermentation. Proc Natl Acad Sci USA.

[17] Kim JW, Chin YW, Park YC, Seo JH (2012). Effects of deletion of glycerol-3-phosphate dehydrogenase and glutamate dehydrogenase genes on glycerol and ethanol metabolism in recombinant *Saccharomyces cerevisiae*. Bioprocess Biosyst Eng.

[18] Demeke M, Dumortier F, Li Y, Broeckx T, Foulquie-Moreno M, Thevelein J (2013). Combining inhibitor tolerance and d-xylose fermentation in industrial *Saccharomyces cerevisiae* for efficient lignocellulose-based bioethanol production. Biotechnol Biofuels.

[19] Diao L, Liu Y, Qian F, Yang J, Jiang Y, Yang S (2013). Construction of fast xylose-fermenting yeast based on industrial ethanol-producing diploid *Saccharomyces cerevisiae* by rational design and adaptive evolution. BMC Biotechnol.

[20] Mortimer RK, Johnston JR (1986). Genealogy of principal strains of the yeast genetic stock center. Genetics.

[21] Wohlbach DJ, Kuo A, Sato TK, Potts KM, Salamov AA (2011). Comparative genomics of xylose-fermenting fungi for enhanced biofuel production. Proc Natl Acad Sci USA.

[22] Wohlbach DJ, Rovinskiy N, Lewis JA, Sardi M, Schackwitz WS, Martin JA, Deshpande S, Daum CG, Lipzen A, Sato TK, Gasch AP (2014). Comparative genomics of *Saccharomyces cerevisiae* natural isolates for bioenergy production. Genome Biol Evol.

[23] McIlwain SJ, Peris D, Sardi M, Moskvin OV, Zhan F, Myers K, Riley NM, Buzzell A, Parreiras LS, Ong IM (2016). Genome sequence and analysis of a stress-tolerant, wild-derived strain of *Saccharomyces cerevisiae* used in biofuels research. G3.

[24] Sato TK, Liu T, Parreiras LS, Williams DL, Wohlbach DJ, Bice BD, Ong IS, Breuer RJ, Qin L, Busalacchi D (2014). Harnessing genetic diversity in *Saccharomyces cerevisiae* for improved fermentation of xylose in hydrolysates of alkaline hydrogen peroxide pretreated biomass. Appl Environ Microbiol.

[25] Parreiras LS, Breuer RJ, Narasimhan RA, Higbee AJ, La Reau A, Tremaine M, Qin L, Willis LB, Bice BD, Bonfert BL (2014). Engineering and two-stage evolution of a lignocellulosic hydrolysate-tolerant *Saccharomyces cerevisiae* strain for anaerobic fermentation of xylose from AFEX pretreated corn stover. PLoS ONE.

[26] Sato TK, Tremaine M, Parreiras LS, Hebert AS, Myers KS, Higbee AJ, Sardi M, McIlwain SJ, Ong IM, Breuer RJ (2016). Directed evolution reveals unexpected epistatic interactions that alter metabolic regulation and enable anaerobic xylose use by *Saccharomyces cerevisiae*. PLoS Genet.

[27] Rogers JK, Church GM (2016). Multiplexed engineering in biology. Trends Biotechnol.

[28] Scannell DR, Zill OA, Rokas A, Payen C, Dunham MJ, Eisen MB, Rine J, Johnston M, Hittinger CT (2011). The awesome power of yeast evolutionary genetics: new genome sequences and strain resources for the *Saccharomyces* sensu stricto genus. G3.

[29] Hittinger CT (2013). *Saccharomyces* diversity and evolution: a budding model genus. Trends Genet.

[30] Dujon B (2006). Yeasts illustrate the molecular mechanisms of eukaryotic genome evolution. Trends Genet.

[31] Leducq JB, Charron G, Samani P, Dubé AK, Sylvester K, James B, Almeida P, Sampaio JP, Hittinger CT, Bell G, Landry CR (2014). Local climatic adaptation in a widespread microorganism. Proc R Soc Lond B Biol Sci.

[32] Hittinger CT, Gonçalves P, Sampaio JP, Dover J, Johnston M, Rokas A (2010). Remarkably ancient balanced polymorphisms in a multi-locus gene network. Nature.

[33] Peris D, Langdon Q, Moriarty R, Sylvester K, Bontrager M, Charron G, Leducq J, Landry C, Libkind D, Hittinger C (2016). Complex ancestries of lager-brewing hybrids were shaped by standing variation in wild yeast *Saccharomyces eubayanus*. PLoS Genet.

[34] Morales L, Dujon B (2012). Evolutionary role of interspecies hybridization and genetic exchanges in yeasts. Microbiol Mol Biol R.

[35] Tirosh I, Reikhav S, Levy AA, Barkai N (2009). A yeast hybrid provides insight into the evolution of gene expression regulation. Science.

[36] Masneuf I, Hansen J, Groth C, Piskur J, Dubourdieu D (1998). New hybrids between *Saccharomyces* sensu stricto yeast species found among wine and cider production strains. Appl Environ Microbiol.

[37] Casaregola S, Nguyen HV, Lapathitis G, Kotyk A, Gaillardin C (2001). Analysis of the constitution of the beer yeast genome by PCR, sequencing and subtelomeric sequence hybridization. Int J Syst Evol Microbiol.

[38] Lopes MB, Bellon JR, Shirley NJ, Ganter PF (2002). Evidence for multiple interspecific hybridization in *Saccharomyces* sensu stricto species. FEMS Yeast Res.

[39] Le Jeune C, Lollier M, Demuyter C, Erny C, Legras JL, Aigle M, Masneuf-Pomarède I (2007). Characterization of natural hybrids of *Saccharomyces cerevisiae* and *Saccharomyces bayanus* var. *uvarum*. FEMS Yeast Res.

[40] Dunn B, Sherlock G (2008). Reconstruction of the genome origins and evolution of the hybrid lager yeast *Saccharomyces pastorianus*. Genome Res.

[41] González SS, Barrio E, Querol A (2008). Molecular characterization of new natural hybrids between *S. cerevisiae* and *S. kudriavzevii* from brewing. Appl Environ Microbiol.

[42] Peris D, Belloch C, Lopandic K, Álvarez-Pérez JM, Querol A, Barrio E (2012). The molecular characterization of new types of *S. cerevisiae* × *S. kudriavzevii* hybrid yeasts unveils a high genetic diversity. Yeast.

[43] Peris D, Lopes CA, Arias A, Barrio E (2012). Reconstruction of the evolutionary history of *Saccharomyces cerevisiae* × *S. kudriavzevii* hybrids based on multilocus sequence analysis. PLoS ONE.

[44] Peris D, Sylvester K, Libkind D, Gonçalves P, Sampaio JP, Alexander WG, Hittinger CT (2014). Population structure and reticulate evolution of *Saccharomyces eubayanus* and its lager-brewing hybrids. Mol Ecol.

[45] Pérez-Través L, Lopes CA, Querol A, Barrio E (2014). On the complexity of the *Saccharomyces bayanus* taxon: hybridization and potential hybrid speciation. PLoS ONE.

[46] Belloch C, Pérez-Torrado R, González SS, Pérez-Ortin JE, García-Martínez J, Querol A, Barrio E (2009). The chimerical genomes of natural hybrids between *Saccharomyces cerevisiae* and *Saccharomyces kudriavzevii*. Appl Environ Microbiol.

[47] Peris D, Lopes CA, Belloch C, Querol A, Barrio E (2012). Comparative genomics among *Saccharomyces cerevisiae* × *Saccharomyces kudriavzevii* natural hybrid strains isolated from wine and beer reveals different origins. BMC Genomics.

[48] Belloch C, Orlic S, Barrio E, Querol A (2008). Fermentative stress adaptation of hybrids within the *Saccharomyces* sensu stricto complex. Int J Food Microbiol.

[49] Gibson BR, Storgårds E, Krogerus K, Vidgren V (2013). Comparative physiology and fermentation performance of Saaz and Frohberg lager yeast strains and the parental species *Saccharomyces eubayanus*. Yeast.

[50] Baker E, Wang B, Bellora N, Peris D, Hulfachor AB, Koshalek JA, Adams M, Libkind D, Hittinger CT (2015). The genome sequence of *Saccharomyces eubayanus* and the domestication of lager-brewing yeasts. Mol Biol Evol.

[51] Antunovics Z, Nguyen HV, Gaillardin C, Sipiczki M (2005). Gradual genome stabilisation by progressive reduction of the *Saccharomyces uvarum* genome in an interspecific hybrid with *Saccharomyces cerevisiae*. FEMS Yeast Res.

[52] Kunicka-Styczynska A, Rajkowska K (2011). Physiological and genetic stability of hybrids of industrial wine yeasts *Saccharomyces* sensu stricto complex. J Appl Microbiol.

[53] Pérez-Través L, Lopes C, Barrio E, Querol A (2014). Stabilization process in *Saccharomyces* intra and interspecific hybrids in fermentative conditions. Int Microbiol.

[54] Kuehne HA, Murphy HA, Francis CA, Sniegowski PD (2007). Allopatric divergence, secondary contact, and genetic isolation in wild yeast populations. Curr Biol.

[55] Sampaio JP, Gonçalves P (2008). Natural populations of *Saccharomyces kudriavzevii* in Portugal are associated with oak bark and are sympatric with *S. cerevisiae* and *S. paradoxus*. Appl Environ Microbiol.

[56] Liti G, Carter DM, Moses AM, Warringer J, Parts L, James SA, Davey RP, Roberts IN, Burt A, Koufopanou V (2009). Population genomics of domestic and wild yeasts. Nature.

[57] Liti G, Ba A, Blythe M, Muller C, Bergstrom A, Cubillos F, Dafhnis-Calas F, Khoshraftar S, Malla S, Mehta N (2013). High quality de novo sequencing and assembly of the *Saccharomyces arboricolus* genome. BMC Genomics.

[58] Libkind D, Hittinger CT, Valério E, Gonçalves C, Dover J, Johnston M, Gonçalves P, Sampaio JP (2011). Microbe domestication and the identification of the wild genetic stock of lager-brewing yeast. Proc Natl Acad Sci USA.

[59] Charron G, Leducq JB, Bertin C, Dubé AK, Landry CR (2013). Exploring the northern limit of the distribution of *Saccharomyces cerevisiae* and *Saccharomyces paradoxus* in North America. FEMS Yeast Res.

[60] Almeida P, Gonçalves C, Teixeira S, Libkind D, Bontrager M, Masneuf-Pomarède I, Albertin W, Durrens P, Sherman DJ, Marullo P (2014). A Gondwanan imprint on global diversity and domestication of wine and cider yeast *Saccharomyces uvarum*. Nat Commun.

[61] Leducq JB, Landry CR, Aubin-Horth N (2014). Ecological genomics of adaptation and speciation in fungi. Ecological genomics.

[62] Leducq JB, Nielly-Thibault L, Charron G, Eberlein C, Verta JP, Samani P, Sylvester K, Hittinger CT, Bell G, Landry CR (2016). Speciation driven by hybridization and chromosomal plasticity in a wild yeast. Nat Microbiol.

[63] Sylvester K, Wang QM, James B, Mendez R, Hulfachor AB, Hittinger CT (2015). Temperature and host preferences drive the diversification of *Saccharomyces* and other yeasts: a survey and the discovery of eight new yeast species. FEMS Yeast Res.

[64] Tomás-Pejó E, Oliva JM, González A, Ballesteros I, Ballesteros M (2009). Bioethanol production from wheat straw by the thermotolerant yeast *Kluyveromyces marxianus* CECT 10875 in a simultaneous saccharification and fermentation fed-batch process. Fuel.

[65] Bals B, Rogers C, Jin M, Balan V, Dale B (2010). Evaluation of ammonia fibre expansion (AFEX) pretreatment for enzymatic hydrolysis of switchgrass harvested in different seasons and locations. Biotechnol Biofuels.

[66] Nielsen J, Keasling JD (2016). Engineering cellular metabolism. Cell.

[67] Hubmann G, Mathe L, Foulquie-Moreno M, Duitama J, Nevoigt E, Thevelein J (2013). Identification of multiple interacting alleles conferring low glycerol and high ethanol yield in *Saccharomyces cerevisiae* ethanolic fermentation. Biotechnol Biofuels.

[68] Zheng DQ, Liu TZ, Chen J, Zhang K, Li O, Zhu L, Zhao YH, Wu XC, Wang PM (2013). Comparative functional genomics to reveal the molecular basis of phenotypic diversities and guide the genetic breeding of industrial yeast strains. Appl Microbiol Biot.

[69] Piotrowski J, Simpkins S, Li S, Deshpande R, McIlwain S, Ong I, Myers C, Boone C, Andersen R, Hempel JE, Williams CH, Hong CC (2015). Chemical genomic profiling via barcode sequencing to predict compound mode of action. Chemical biology.

[70] Pérez-Través L, Lopes CA, González R, Barrio E, Querol A (2015). Physiological and genomic characterisation of *Saccharomyces cerevisiae* hybrids with improved fermentation performance and mannoprotein release capacity. Int J Food Microbiol.

[71] Hebly M, Brickwedde A, Bolat I, Driessen MRM, de Hulster EAF, van den Broek M, Pronk JT, Geertman JM, Daran JM, Daran-Lapujade P (2015). *S. cerevisiae* × *S. eubayanus* interspecific hybrid, the best of both worlds and beyond. FEMS Yeast Res.

[72] Alexander WG, Peris D, Pfannenstiel BT, Opulente DA, Kuang M, Hittinger CT (2016). Efficient engineering of marker-free synthetic allotetraploids of *Saccharomyces*. Fungal Genet Biol.

[73] Hewitt SK, Donaldson IJ, Lovell SC, Delneri D (2014). Sequencing and characterisation of rearrangements in three *S. pastorianus* strains reveals the presence of chimeric genes and gives evidence of breakpoint reuse. PLoS ONE.

[74] Okuno M, Kajitani R, Ryusui R, Morimoto H, Kodama Y, Itoh T (2016). Next-generation sequencing analysis of lager brewing yeast strains reveals the evolutionary history of interspecies hybridization. DNA Res.

[75] Kodama Y, Kielland-Brandt MC, Hansen J, Sunnerhagen P, Piskur J (2005). Lager brewing yeast. Comparative Genomics: using fungi as models.

[76] Koch MA, Dobeš C, Mitchell-Olds T (2003). Multiple hybrid formation in natural populations: concerted evolution of the Internal Transcribed Spacer of nuclear ribosomal DNA (ITS) in North American *Arabis divaricarpa* (Brassicaceae). Mol Biol Evol.

[77] Sebastiani F, Barberio C, Casalone E, Cavalieri D, Polsinelli M (2002). Crosses between *Saccharomyces cerevisiae* and *Saccharomyces bayanus* generate fertile hybrids. Res Microbiol.

[78] Sipiczki M (2008). Interspecies hybridization and recombination in *Saccharomyces* wine yeasts. FEMS Yeast Res.

[79] Field S, Ryden P, Wilson D, James S, Roberts I, Richardson D, Waldron K, Clarke T (2015). Identification of furfural resistant strains of *Saccharomyces cerevisiae* and *Saccharomyces paradoxus* from a collection of environmental and industrial isolates. Biotechnol Biofuels.

[80] Wimalasena TT, Greetham D, Marvin ME, Liti G, Chandelia Y, Hart A, Louis EJ, Phister TG, Tucker GA, Smart KA (2014). Phenotypic characterisation of *Saccharomyces* spp. yeast for tolerance to stresses encountered during fermentation of lignocellulosic residues to produce bioethanol. Microb Cell Fact.

[81] Kurtzman CP, Fell JW, Boekhout T (2011). The yeasts: a taxonomic study.

[82] Attfield PV, Bell PJL (2006). Use of population genetics to derive nonrecombinant *Saccharomyces cerevisiae* strains that grow using xylose as a sole carbon source. FEMS Yeast Res.

[83] Wenger JW, Schwartz K, Sherlock G (2010). Bulk segregant analysis by high-throughput sequencing reveals a novel xylose utilization gene from *Saccharomyces cerevisiae*. PLoS Genet.

[84] Dashtban M, Schraft H, Syed TA, Qin W (2010). Fungal biodegradation and enzymatic modification of lignin. Int J Biochem Mol Biol.

[85] Medina A, Jakobsen I, Egsgaard H (2011). Sugar beet waste and its component ferulic acid inhibits external mycelium of arbuscular mycorrhizal fungus. Soil Biol Biochem.

[86] Vladimir U, Pawel J, Grillo O, Venora G (2011). Biodiversity of yeasts in the gastrointestinal ecosystem with emphasis on its importance for the host. The dynamical processes of biodiversity—case studies of evolution and spatial distribution.

[87] Selmecki AM, Maruvka YE, Richmond PA, Guillet M, Shoresh N, Sorenson AL, De S, Kishony R, Michor F, Dowell R, Pellman D (2015). Polyploidy can drive rapid adaptation in yeast. Nature.

[88] Alexander WG, Doering DT, Hittinger CT (2014). High-efficiency genome editing and allele replacement in prototrophic and wild strains of *Saccharomyces*. Genetics.

[89] Wang H, Qi M, Cutler AJ (1993). A simple method of preparing plant samples for PCR. Nucleic Acids Res.

[90] Belloch C, Querol A, Garcia MD, Barrio E (2000). Phylogeny of the genus *Kluyveromyces* inferred from the mitochondrial cytochrome-c oxidase II gene. Int J Syst Evol Microbiol.

[91] Bukhman Y, DiPiazza N, Piotrowski J, Shao J, Halstead A, Bui M, Xie E, Sato T (2015). Modeling microbial growth curves with GCAT. Bioenerg Res.

[92] R Development Core Team (2010). R: a language and environment for statistical computing.

[93] Schwalbach MS, Keating DH, Tremaine M, Marner WD, Zhang Y, Bothfeld W, Higbee A, Grass JA, Cotten C, Reed JL (2012). Complex physiology and compound stress responses during fermentation of alkali-pretreated corn stover hydrolysate by an *Escherichia coli* ethanologen. Appl Environ Microbiol.

[94] Salvadó Z, Arroyo-Lopez FN, Guillamón JM, Salazar G, Querol A, Barrio E (2011). Temperature adaptation markedly determines evolution within the genus *Saccharomyces*. Appl Environ Microbiol.

[95] Bolger AM, Lohse M, Usadel B (2014). Trimmomatic: a flexible trimmer for Illumina sequence data. Bioinformatics.

[96] Bankevich A, Nurk S, Antipov D, Gurevich AA, Dvorkin M, Kulikov AS, Lesin VM, Nikolenko SI, Pham S, Prjibelski AD (2012). SPAdes: a new genome assembly algorithm and its applications to single-cell sequencing. J Comput Biol.

[97] Zhou X, Peris D, Kominek J, Kurtzman CP, Hittinger CT, Rokas A (2016). In silico Whole Genome Sequencer & Analyzer (iWGS): a computational pipeline to guide the design and analysis of de novo genome sequencing studies. G3.

[98] Kearse M, Moir R, Wilson A, Stones-Havas S, Cheung M, Sturrock S, Buxton S, Cooper A, Markowitz S, Duran C, Thierer T, Ashton B, Meintjes P, Drummond A (2012). Geneious Basic: an integrated and extendable desktop software platform for the organization and analysis of sequence data. Bioinformatics.

[99] Teytelman L, Özaydin B, Zill O, Lefrançois P, Snyder M, Rine J, Eisen MB (2009). Impact of chromatin structures on DNA processing for genomic analyses. PLoS ONE.

[100] Peris D, Arias A, Orlic S, Belloch C, Perez-Traves L, Querol A, Barrio E (2017). Mitochondrial introgression suggests extensive ancestral hybridization events among *Saccharomyces* species. Mol Phylogenet Evol.

[101] Nakao Y, Kanamori T, Itoh T, Kodama Y, Rainieri S, Nakamura N, Shimonaga T, Hattori M, Ashikari T (2009). Genome sequence of the lager brewing yeast, an interspecies hybrid. DNA Res.

