# Supplementary material for: Hybridization and adaptive evolution of diverse Saccharomyces species for cellulosic biofuel production
Source: Biotechnol Biofuels. 2017 Mar 27;10:78. doi: 10.1186/s13068-017-0763-7 (PMC5369230; doi:10.1186/s13068-017-0763-7)
Supplement: Supplementary file 3 — Additional file 3. Maximum growth rate heatmap of two ancestral and two evolved synthetic hybrids and the GLBRCY73 strain. A) The average values (n = 2) of maximum growth rate (µ, defined as (ln(OD2)-ln(OD1))/(T2-T1)) in different media conditions at 30 °C are shown. Heat colors from yellow (low growth rate) to blue (high growth rate) are scaled according to left bar. Media conditions are clustered by Euclidean distance. Sc: S. cerevisiae, Sm: S. mikatae, Sk: S. kudriavzevii. [file 13068_2017_763_MOESM3_ESM.pptx]

## Slide 1
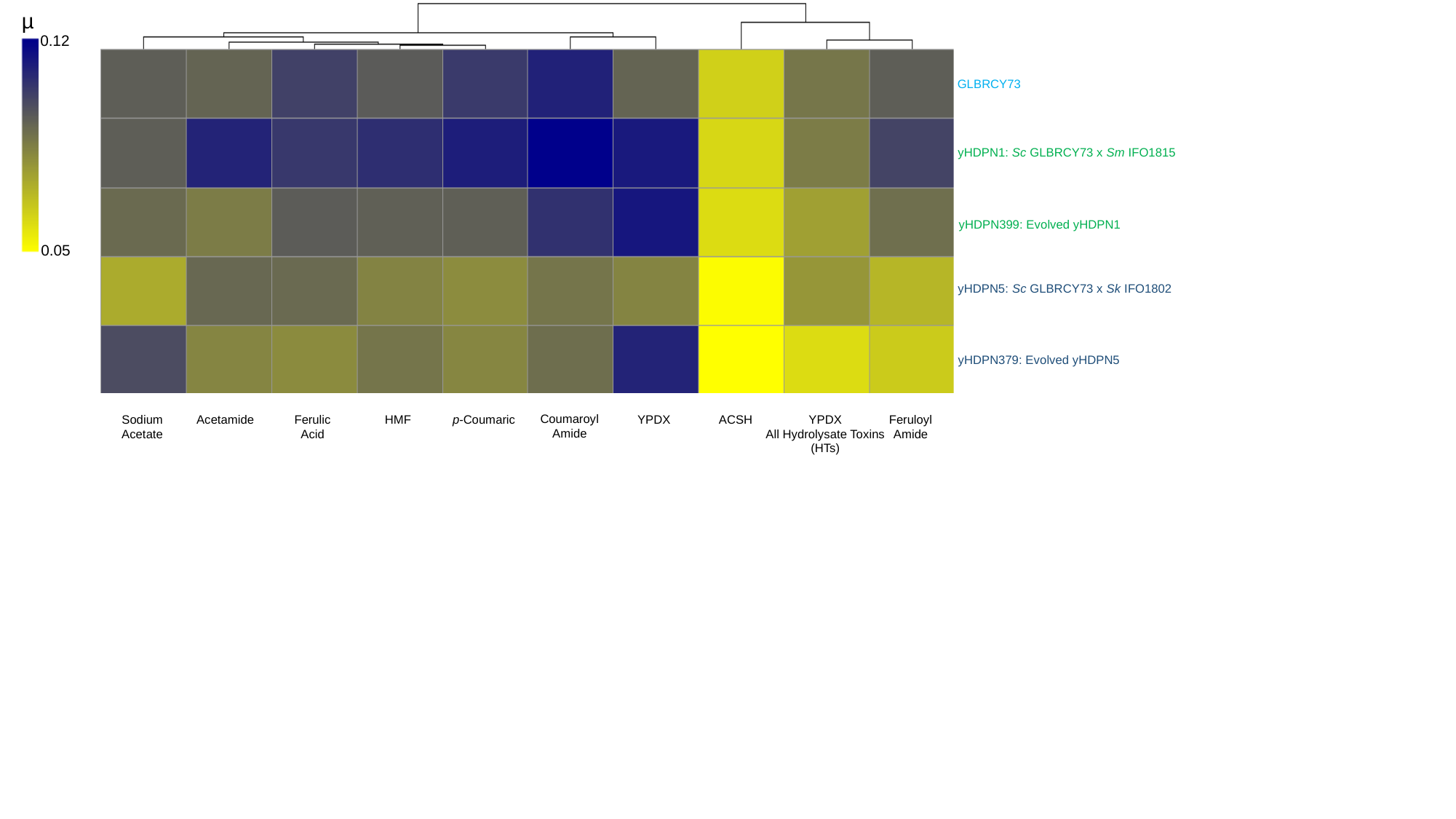

µ
0.12
GLBRCY73
yHDPN1: Sc GLBRCY73 x Sm IFO1815
yHDPN399: Evolved yHDPN1
0.05
yHDPN5: Sc GLBRCY73 x Sk IFO1802
yHDPN379: Evolved yHDPN5
Coumaroyl
Amide
YPDX
All Hydrolysate Toxins
(HTs)
Acetamide
HMF
p-Coumaric
Sodium
Acetate
ACSH
Ferulic
Acid
YPDX
Feruloyl
Amide
