# Supplementary material for: Hybridization and adaptive evolution of diverse Saccharomyces species for cellulosic biofuel production
Source: Biotechnol Biofuels. 2017 Mar 27;10:78. doi: 10.1186/s13068-017-0763-7 (PMC5369230; doi:10.1186/s13068-017-0763-7)
Supplement: Supplementary file 5 — Additional file 5. Ethanol yield (%) for ancestral synthetic hybrids and evolved synthetic hybrids during the xylose fermentation phase of the culture. Bar plots represent the difference between the percentage of ethanol yield at day seven and day 2, the point at which all glucose had been consumed. Panel A and B represent the values for S. cerevisiae × S. mikatae and S. cerevisiae × S. kudriavzevii synthetic hybrids, respectively. S. cer, S. cerevisiae; S. mik, S. mikatae; S. kud, S. kudriavzevii. Colors are the values for each condition according to the legend. ACSH-Anaer, ACSH anaerobic; ACSH-Micro, ACSH microaerobic; YPDX + HTs, YPDX + Hydrolysate toxins cocktail; YPDX + FA, YPDX + Feruloyl Amide. The p-values from t-tests are represented by * when < 0.05, ** < 0.01, *** < 0.001. [file 13068_2017_763_MOESM5_ESM.pptx]

## Slide 1
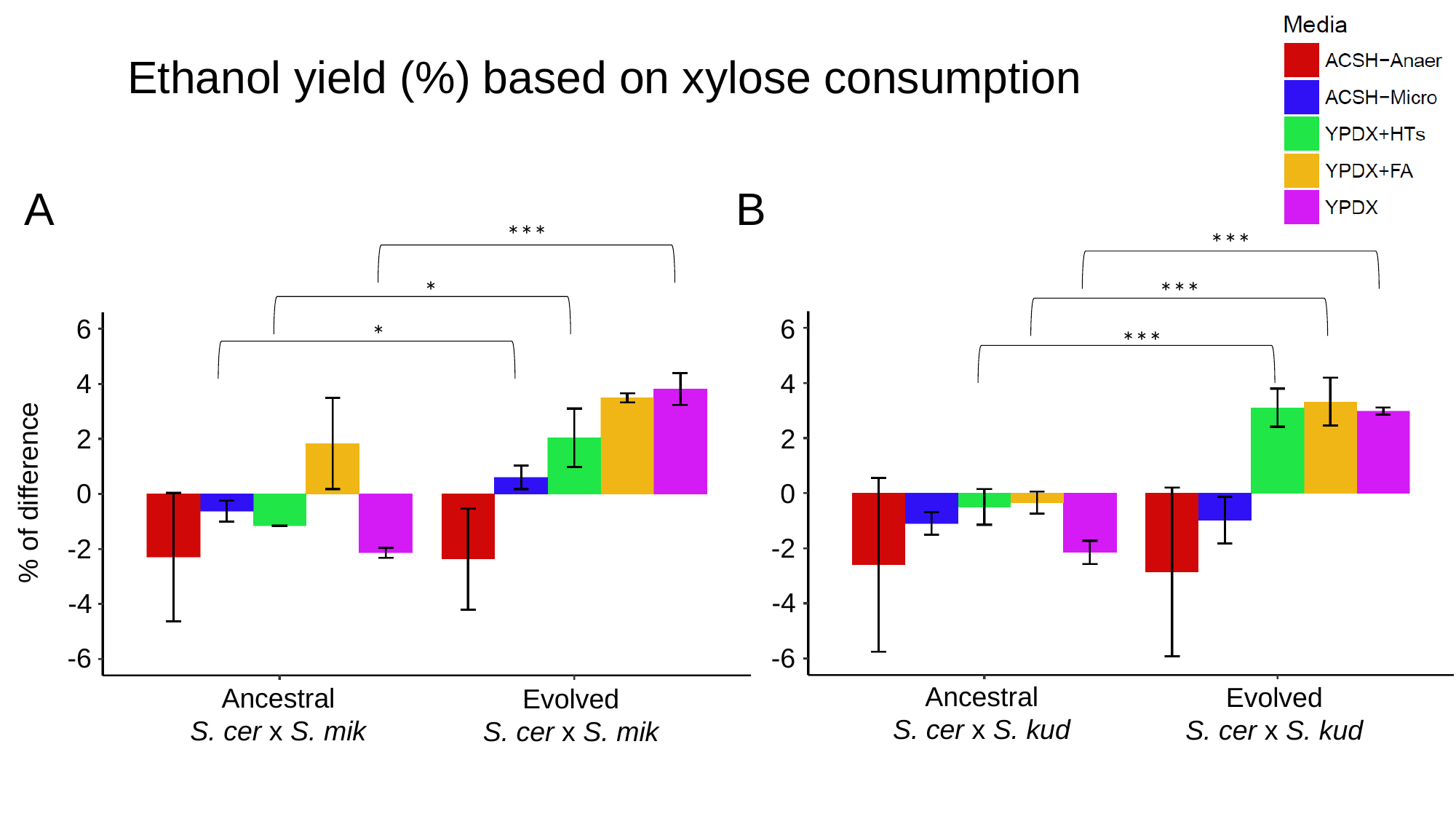

Ethanol yield (%) based on xylose consumption
A
B
***
***
*
***
6
6
*
***
4
4
2
2
% of difference
0
0
-2
-2
-4
-4
-6
-6
Ancestral
S. cer x S. kud
Evolved
S. cer x S. kud
Ancestral
S. cer x S. mik
Evolved
S. cer x S. mik
