# Supplementary material for: Hybridization and adaptive evolution of diverse Saccharomyces species for cellulosic biofuel production
Source: Biotechnol Biofuels. 2017 Mar 27;10:78. doi: 10.1186/s13068-017-0763-7 (PMC5369230; doi:10.1186/s13068-017-0763-7)
Supplement: Supplementary file 7 — Additional file 7. Compound time course curves for GLBRCY73, synthetic ancestral hybrids, and evolved hybrids of S. cerevisiae × S. kudriavzevii. Panels A-E represent the extracellular concentration (g/L) of glucose, xylose, and ethanol through the fermentation of various media in various conditions for S. cerevisiae × S. kudriavzevii. Panels F-J represent the optical density at 600 nm variation through the aforementioned fermentations. S. cer, S. cerevisiae; S. kud, S. kudriavzevii. [file 13068_2017_763_MOESM7_ESM.pptx]

## Slide 1
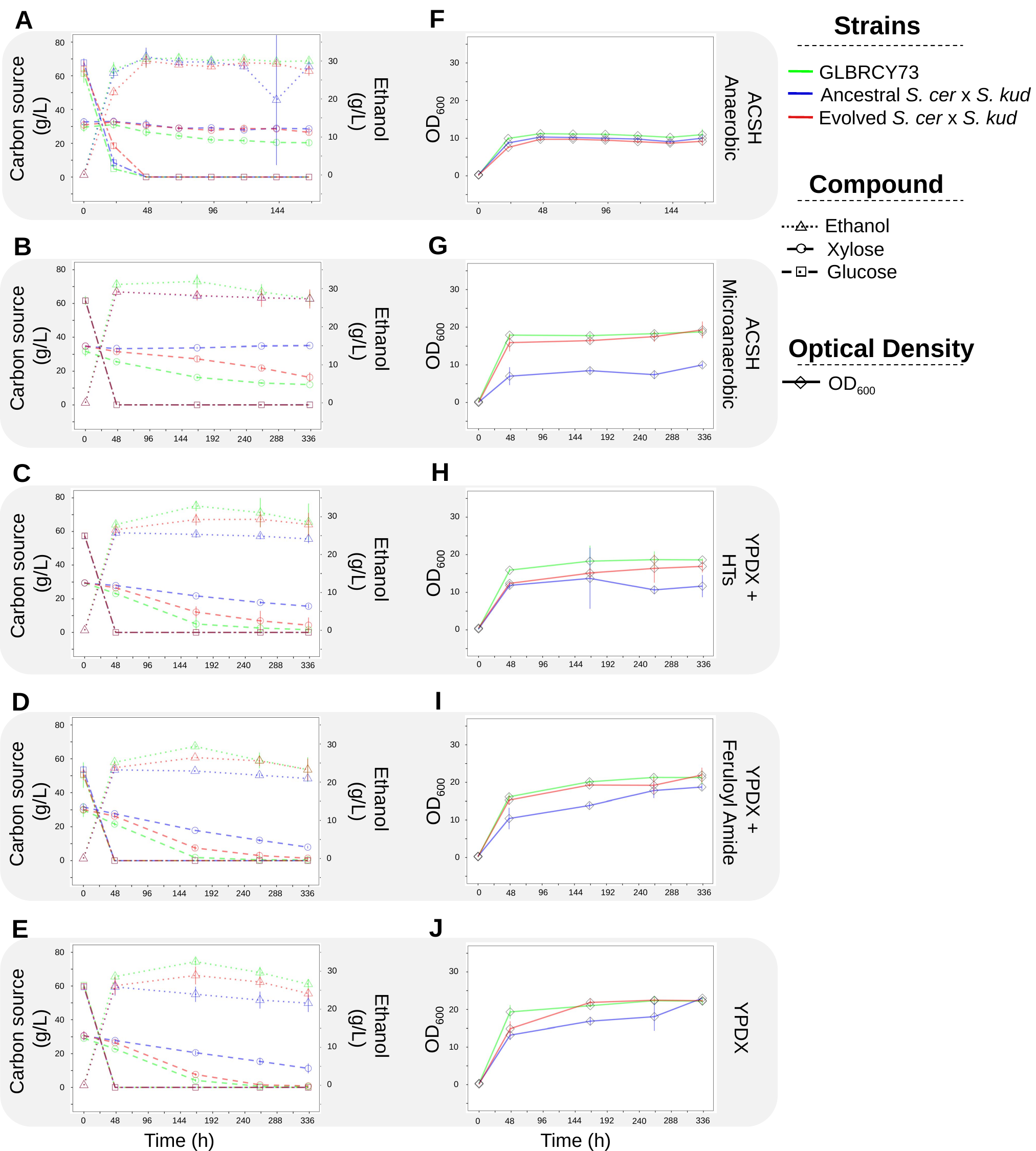

F
A
Strains
80
30
30
GLBRCY73
60
Ancestral S. cer x S. kud
Ethanol
 (g/L)
20
20
ACSH
Anaerobic
Carbon source
 (g/L)
40
Evolved S. cer x S. kud
OD600
10
10
20
Compound
0
0
0
144
96
144
48
96
48
0
0
Ethanol
G
B
Xylose
Glucose
80
30
30
60
Ethanol
 (g/L)
ACSH
Microanaerobic
20
20
Carbon source
 (g/L)
40
Optical Density
OD600
10
10
20
OD600
0
0
0
288
336
192
144
96
0
240
48
288
336
192
144
96
0
240
48
H
C
80
30
30
60
Ethanol
 (g/L)
YPDX +
HTs
20
20
Carbon source
 (g/L)
40
OD600
10
10
20
0
0
0
288
336
192
144
96
0
240
48
288
336
192
144
96
0
240
48
I
D
80
30
30
60
Ethanol
 (g/L)
YPDX +
 Feruloyl Amide
20
20
Carbon source
 (g/L)
40
OD600
10
10
20
0
0
0
288
336
192
144
96
0
240
48
288
336
192
144
96
0
240
48
J
E
80
30
30
60
Ethanol
 (g/L)
20
20
Carbon source
 (g/L)
40
YPDX
OD600
10
10
20
0
0
0
288
336
192
144
96
0
240
48
288
336
192
144
96
0
240
48
Time (h)
Time (h)
