# Supplementary material for: Hybridization and adaptive evolution of diverse Saccharomyces species for cellulosic biofuel production
Source: Biotechnol Biofuels. 2017 Mar 27;10:78. doi: 10.1186/s13068-017-0763-7 (PMC5369230; doi:10.1186/s13068-017-0763-7)
Supplement: Supplementary file 8 — Additional file 8. Flow cytometry fluorescence intensity distribution for reference strains and synthetic hybrids. Y73 is a diploid strain [21, 24], and W34/70 is a hybrid between S. cerevisiae and S. eubayanus that is approximately tetraploid [101]. By comparing the cell count distribution of fluorescence intensity of SYBR green of each strain, we infer strains yHDPN1, yHDPN5, and yHDPN399 to be approximately diploid (2n). The yHDPN379 distribution is between Y73 and W34/70 distribution, suggesting that it is approximately triploid (3n). Scer: S. cerevisiae, Smik: S. mikatae, Skud: S. kudriavzevii, Seub: S. eubayanus. A.U.: Arbitrary Units. [file 13068_2017_763_MOESM8_ESM.pptx]

## Slide 1
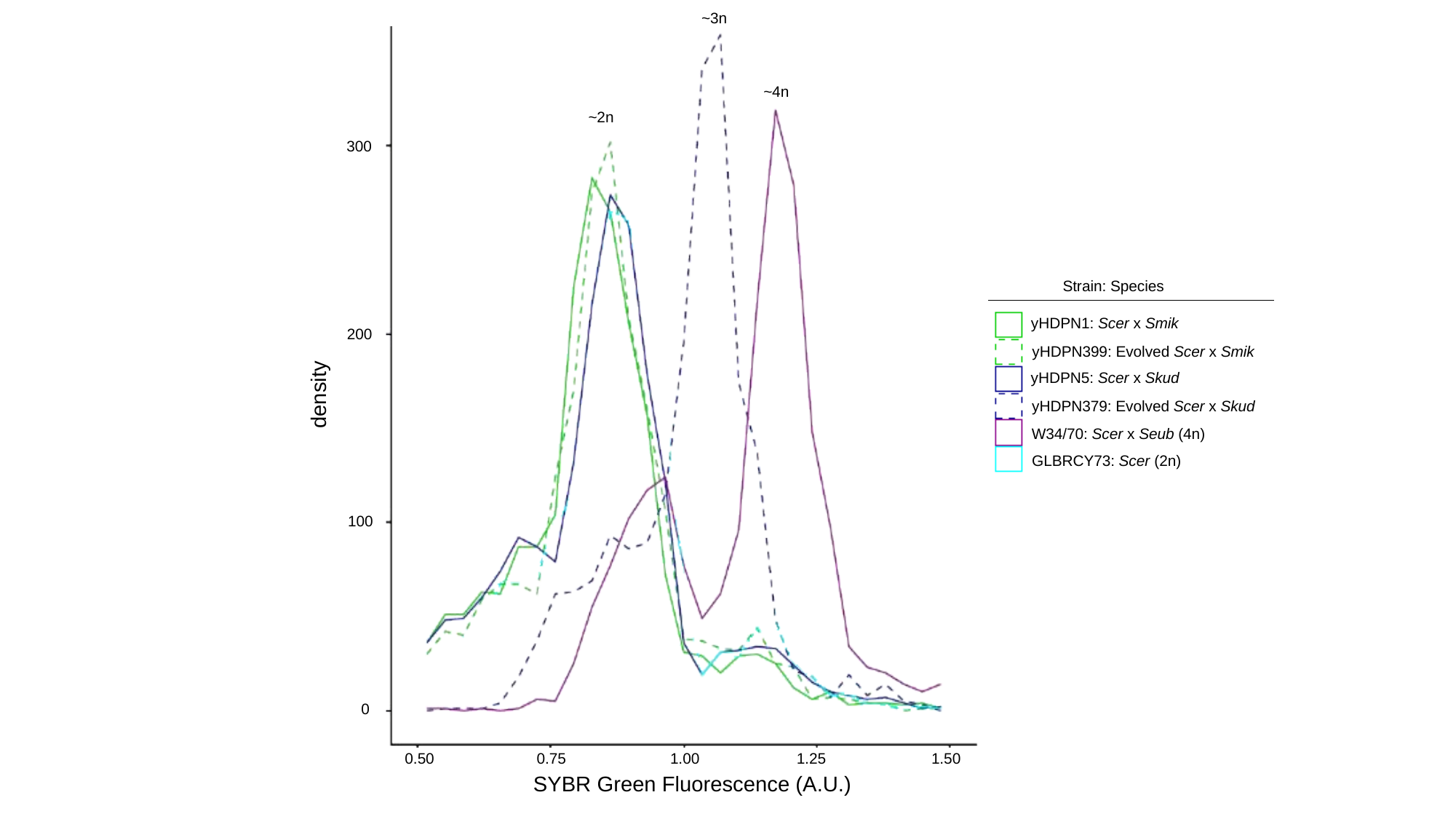

~3n
~4n
~2n
300
Strain: Species
yHDPN1: Scer x Smik
200
yHDPN399: Evolved Scer x Smik
yHDPN5: Scer x Skud
density
yHDPN379: Evolved Scer x Skud
W34/70: Scer x Seub (4n)
GLBRCY73: Scer (2n)
100
0
1.00
1.25
1.50
0.50
0.75
SYBR Green Fluorescence (A.U.)
