# Supplementary material for: Hybridization and adaptive evolution of diverse Saccharomyces species for cellulosic biofuel production
Source: Biotechnol Biofuels. 2017 Mar 27;10:78. doi: 10.1186/s13068-017-0763-7 (PMC5369230; doi:10.1186/s13068-017-0763-7)
Supplement: Supplementary file 10 — Additional file 10. Normalized maximum growth rate, maximum OD595, and lag time boxplots by populations. The average values (n = 2) of normalized maximum growth rate (µ, defined as (ln(OD2)-ln(OD1))/(T2-T1)), maximum OD595, and lag time (adaptation time, h) for strains designated in lineages (Additional File 5) are shown. Median values for each lineage are represented by a black horizontal line inside the box, and the upper and lower whiskers represent the highest and lowest values of the 1.5 * IQR (inter-quartile range), respectively. Letters are Dunn’s test homogeneous groups inferred after pairwise comparisons. Colored lines highlight the values for the type strains, which were used to generate the synthetic hybrids. [file 13068_2017_763_MOESM10_ESM.pptx]

## Slide 1
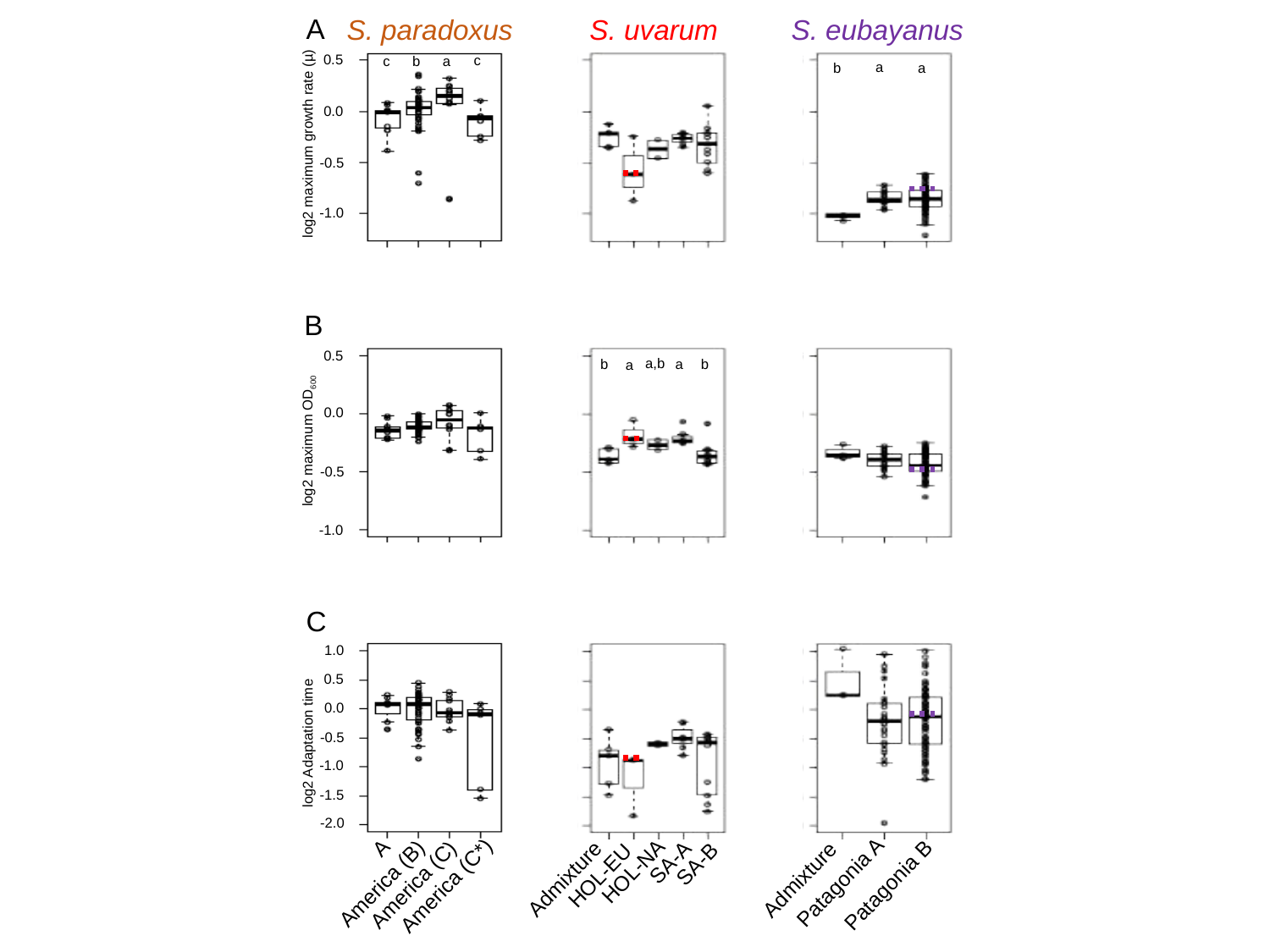

A
S. paradoxus
S. uvarum
S. eubayanus
0.5
c
a
c
b
a
b
a
0.0
log2 maximum growth rate (µ)
-0.5
-1.0
B
0.5
a,b
b
b
a
a
0.0
log2 maximum OD600
-0.5
-1.0
C
1.0
0.5
0.0
-0.5
log2 Adaptation time
-1.0
-1.5
-2.0
A
SA-A
SA-B
HOL-NA
HOL-EU
Admixture
Admixture
Patagonia A
America (B)
America (C)
Patagonia B
America (C*)
