# Supplementary material for: Hybridization and adaptive evolution of diverse Saccharomyces species for cellulosic biofuel production
Source: Biotechnol Biofuels. 2017 Mar 27;10:78. doi: 10.1186/s13068-017-0763-7 (PMC5369230; doi:10.1186/s13068-017-0763-7)
Supplement: Supplementary file 11 — Additional file 11. Maximum specific growth rates for each species and synthetic hybrids in specific media conditions. The average values (n = 2) of maximum growth rate (µ, defined as (ln(OD2)-ln(OD1))/(T2-T1)), from data represented in Fig. 6 but categorized by each condition and by species, are shown as boxplots. Letters are Dunn’s test homogeneous groups inferred from pairwise comparisons. Colored boxplots and data points are according to the legend. Median values for each population are represented by a horizontal line inside the box, and the upper and lower whiskers represent the highest and lowest values of the 1.5 * IQR (inter-quartile range), respectively. HTs: hydrolysate toxins. [file 13068_2017_763_MOESM11_ESM.pptx]

## Slide 1
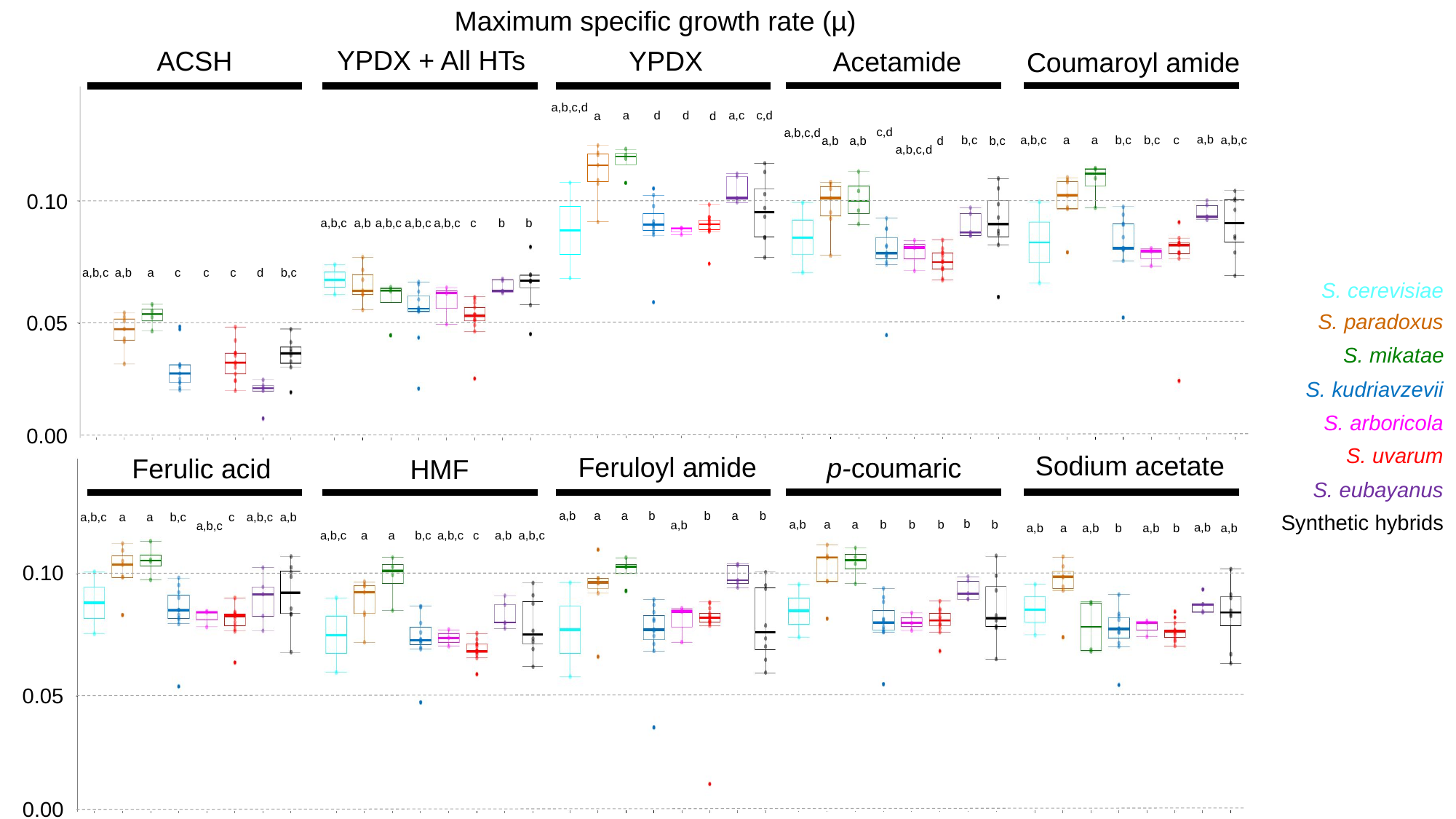

Maximum specific growth rate (µ)
YPDX + All HTs
YPDX
ACSH
Acetamide
Coumaroyl amide
a,b,c,d
a,c
d
d
a
c,d
a
d
c,d
a,b,c,d
a,b
b,c
a,b,c
b,c
a
a,b,c
a
c
b,c
a,b
b,c
a,b
d
a,b,c,d
0.10
b
a,b,c
a,b,c
a,b,c
a,b,c
b
a,b
c
d
c
a,b,c
c
a
b,c
a,b
c
S. cerevisiae
S. paradoxus
0.05
S. mikatae
S. kudriavzevii
S. arboricola
0.00
S. uvarum
Sodium acetate
Feruloyl amide
p-coumaric
Ferulic acid
HMF
S. eubayanus
a
a,b
b
a
b
a
b
Synthetic hybrids
a,b,c
a,b,c
b,c
a
a,b
a
c
b
b
a,b
b
a
b
a
b
a,b
a,b,c
a,b
a,b
a,b
b
a,b
a
b
a,b
a,b
a,b,c
a,b,c
b,c
a
a,b,c
a
c
0.10
0.05
0.00
