# Supplementary material for: Hybridization and adaptive evolution of diverse Saccharomyces species for cellulosic biofuel production
Source: Biotechnol Biofuels. 2017 Mar 27;10:78. doi: 10.1186/s13068-017-0763-7 (PMC5369230; doi:10.1186/s13068-017-0763-7)
Supplement: Supplementary file 12 — Additional file 12. Pairwise correlation heatmaps for each media tested at 24 °C. A), B), and C) represent the pairwise Spearman correlation heatmaps among media tested in Fig. 6. Heat colors represent the degree of correlation from blue (low correlation among strain response, 0) to red (high correlation among strain response, 1). HTs: hydrolysate toxins. µ: (ln(OD2)-ln(OD1))/(T2-T1). [file 13068_2017_763_MOESM12_ESM.pptx]

## Slide 1
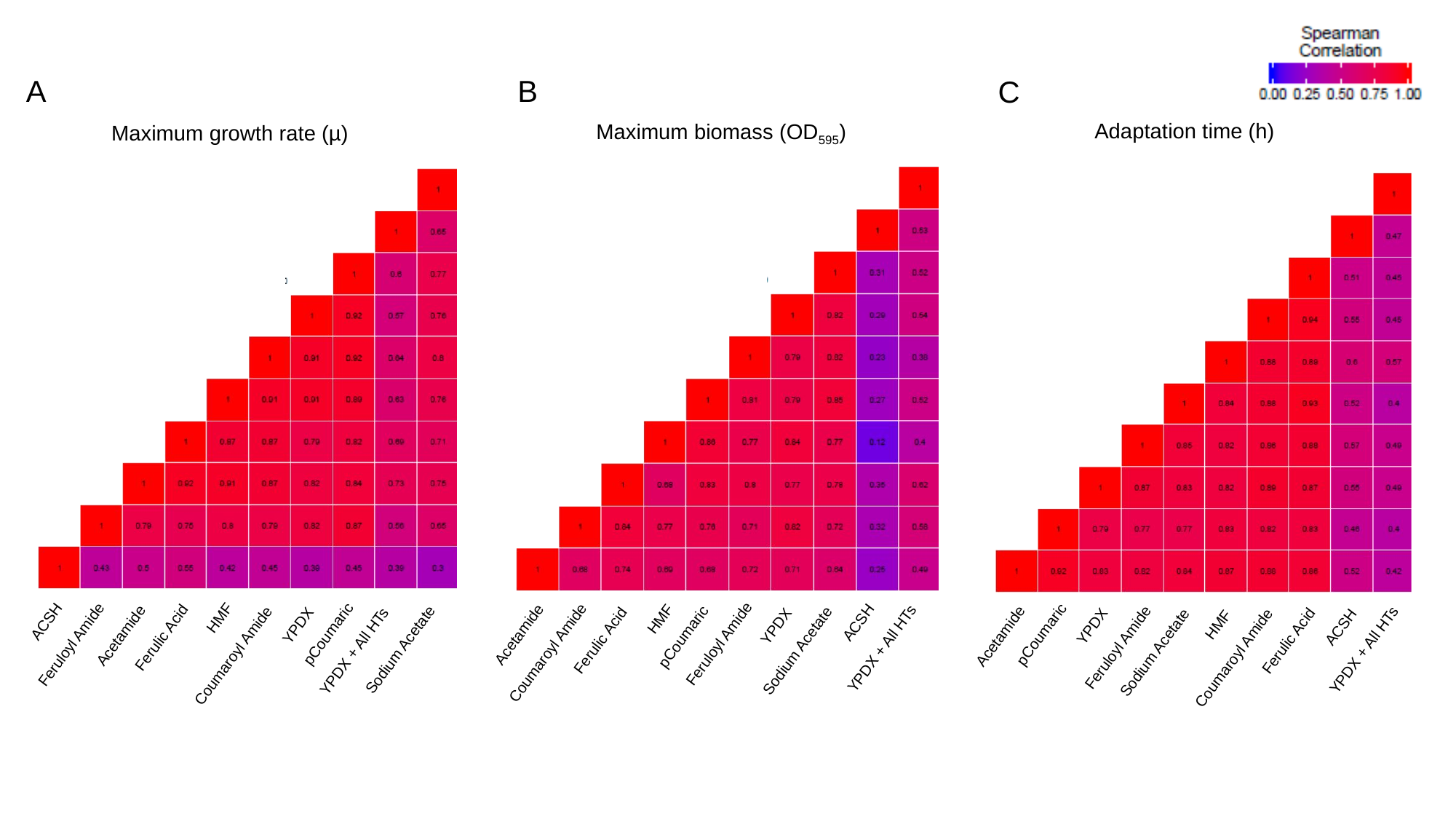

A
B
C
Adaptation time (h)
Maximum biomass (OD595)
Maximum growth rate (µ)
HMF
HMF
ACSH
ACSH
HMF
YPDX
YPDX
YPDX
ACSH
pCoumaric
pCoumaric
Acetamide
Acetamide
Acetamide
pCoumaric
Ferulic Acid
Ferulic Acid
Ferulic Acid
Feruloyl Amide
Feruloyl Amide
Feruloyl Amide
YPDX + All HTs
Sodium Acetate
YPDX + All HTs
Sodium Acetate
YPDX + All HTs
Sodium Acetate
Coumaroyl Amide
Coumaroyl Amide
Coumaroyl Amide
