# Supplementary material for: Hybridization and adaptive evolution of diverse Saccharomyces species for cellulosic biofuel production
Source: Biotechnol Biofuels. 2017 Mar 27;10:78. doi: 10.1186/s13068-017-0763-7 (PMC5369230; doi:10.1186/s13068-017-0763-7)
Supplement: Supplementary file 13 — Additional file 13. Compound time course curves for engineered S. cerevisiae and wild Saccharomyces strains. Panels A-H represent the extracellular concentration (g/L) of glucose, xylose, and ethanol through microaerobic ACSH fermentation by different engineered and wild Saccharomyces strains. Panels I-P represent the variation of the optical density at 600 nm through the aforementioned fermentations. [file 13068_2017_763_MOESM13_ESM.pptx]

## Slide 1
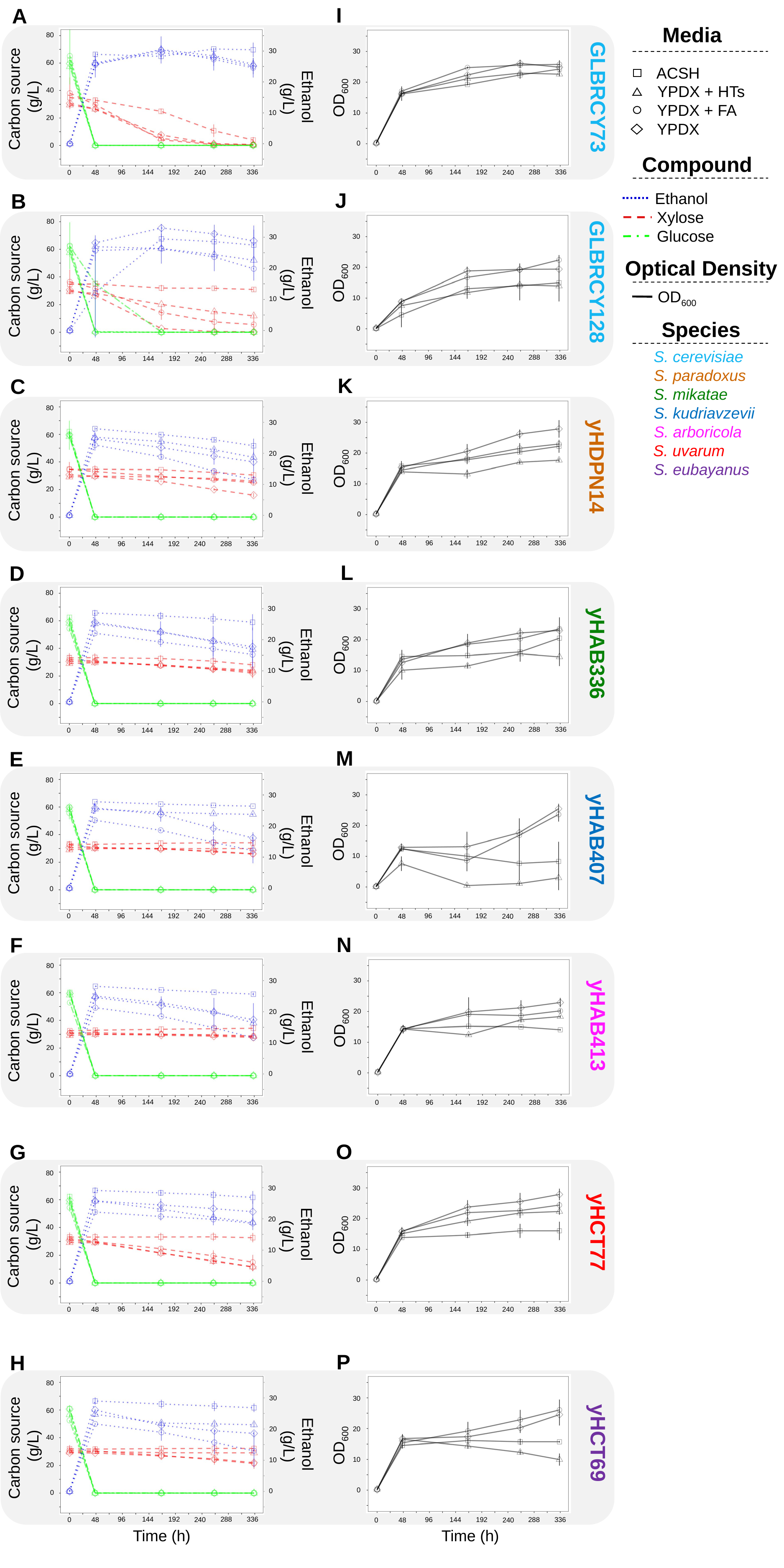

I
A
Media
80
30
30
60
ACSH
20
20
Ethanol
 (g/L)
Carbon source
 (g/L)
YPDX + HTs
GLBRCY73
40
OD600
YPDX + FA
10
10
20
YPDX
0
0
0
Compound
288
336
192
288
336
144
96
192
144
96
0
240
0
240
48
48
J
B
Ethanol
Xylose
80
Glucose
30
30
60
Optical Density
20
20
Ethanol
 (g/L)
Carbon source
 (g/L)
GLBRCY128
40
OD600
OD600
10
10
20
Species
0
0
0
S. cerevisiae
288
336
192
144
96
0
240
48
288
336
192
144
96
0
240
48
S. paradoxus
K
C
S. mikatae
S. kudriavzevii
80
30
30
S. arboricola
60
S. uvarum
20
20
Ethanol
 (g/L)
Carbon source
 (g/L)
yHDPN14
40
OD600
S. eubayanus
10
10
20
0
0
0
288
336
192
144
96
0
240
48
288
336
192
144
96
0
240
48
L
D
80
30
30
60
20
20
Ethanol
 (g/L)
Carbon source
 (g/L)
yHAB336
40
OD600
10
10
20
0
0
0
288
336
192
144
96
0
240
48
288
336
192
144
96
0
240
48
M
E
80
30
30
60
20
20
Ethanol
 (g/L)
Carbon source
 (g/L)
yHAB407
40
OD600
10
10
20
0
0
0
288
336
192
144
96
0
240
48
288
336
192
144
96
0
240
48
N
F
80
30
30
60
20
20
Ethanol
 (g/L)
Carbon source
 (g/L)
yHAB413
40
OD600
10
10
20
0
0
0
288
336
192
144
96
0
240
48
288
336
192
144
96
0
240
48
O
G
80
30
30
60
20
20
Ethanol
 (g/L)
Carbon source
 (g/L)
yHCT77
40
OD600
10
10
20
0
0
0
288
336
192
144
96
0
240
48
288
336
192
144
96
0
240
48
P
H
80
30
30
60
20
20
Ethanol
 (g/L)
Carbon source
 (g/L)
yHCT69
40
OD600
10
10
20
0
0
0
288
336
192
144
96
0
240
48
288
336
192
144
96
0
240
48
Time (h)
Time (h)
