# Supplementary material for: Hybridization and adaptive evolution of diverse Saccharomyces species for cellulosic biofuel production
Source: Biotechnol Biofuels. 2017 Mar 27;10:78. doi: 10.1186/s13068-017-0763-7 (PMC5369230; doi:10.1186/s13068-017-0763-7)
Supplement: Supplementary file 14 — Additional file 14. Compound dynamics through fermentation in different media conditions. Averages and standard deviations of D-xylose, xylitol, glycerol, ethanol concentration, as well as biomass production during the fermentations reported in Fig. 7 are shown in A), B), C), D), and E) graph plots, respectively. Colored lines represent the media condition according to the legend. FA: ferulic acid, HTs: hydrolysate toxins. [file 13068_2017_763_MOESM14_ESM.pptx]

## Slide 1
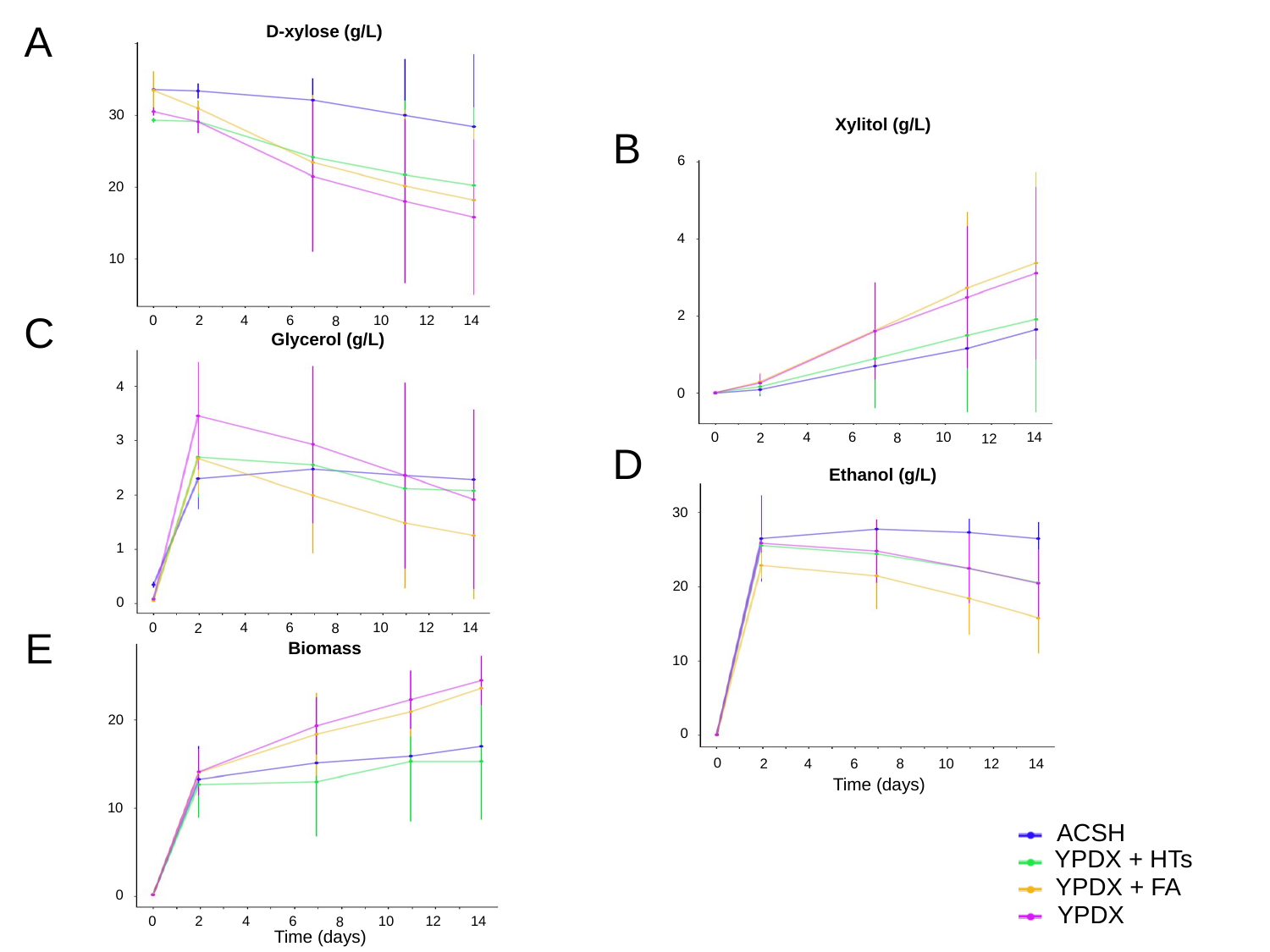

A
D-xylose (g/L)
30
Xylitol (g/L)
B
6
20
4
10
2
C
14
12
6
4
0
10
2
8
Glycerol (g/L)
4
0
14
6
4
0
10
2
8
12
3
D
Ethanol (g/L)
2
30
1
20
0
14
12
6
4
0
10
2
8
E
Biomass
10
20
0
0
8
2
10
14
12
6
4
Time (days)
10
ACSH
YPDX + HTs
YPDX + FA
0
YPDX
12
6
4
0
10
2
14
8
Time (days)
